# Strategies to Reduce the Euthanasia of Impounded Dogs and Cats Used by Councils in Victoria, Australia

**DOI:** 10.3390/ani8070100

**Published:** 2018-06-21

**Authors:** Jacquie Rand, Emily Lancaster, Georgina Inwood, Carolyn Cluderay, Linda Marston

**Affiliations:** 1 School of Veterinary Science, The University of Queensland, Gatton, QLD 4343, Australia; georgina.inwood@uqconnect.edu.au (G.I.); carolyn.cluderay@uq.edu.au (C.C.); 2 Australian Pet Welfare Foundation, Kenmore, QLD 4069, Australia; emily.lancaster1@uqconnect.edu.au (E.L.); linda@marston.com.au (L.M.)

**Keywords:** council pound, dog, cat, euthanasia, Australia

## Abstract

**Simple Summary:**

Euthanasia is used in developed countries as a method of population control for dogs and cats entering shelters and council pounds. This study analyzed all available dog and cat population, registration, intake and outcome data for the 79 Victorian councils. The majority (74%) of councils achieved euthanasia rates for dogs of ≤10%, but only one achieved that for cats, and mean cat euthanasia was 48%. Low euthanasia rates were associated with high rates of reclaim, and adoption of unclaimed animals. A telephone survey of 35 councils (44%) was undertaken to identify policies, practices and attitudes of staff to identify strategies that reduce euthanasia. It is envisaged this data could be used as a resource for councils to lower euthanasia rates.

**Abstract:**

Using euthanasia to manage dog and cat overpopulation causes health issues and emotional stress in employees involved, increases staff turnover, and has financial, moral and ethical ramifications for communities. Welfare agencies and local government agencies (councils) share responsibility for managing companion animal populations. This study investigated Australian councils in the state of Victoria, to identify strategies used to reduce euthanasia. Statistics regarding animal populations, registration, intake, reclaim, rehome and euthanasia were obtained from the Domestic Animal Management Plan of each council. Of the 79 Victorian councils, 74% achieved ≤10% euthanasia of impounded dogs, which is widely quoted as zero euthanasia of adoptable and treatable animals. The mean euthanasia rates for cats by the councils was 48%, with only one council achieving a euthanasia rate of ≤10% for cats. Mean reclaim rates for dogs were higher (73%) than for cats (13%), as was the mean proportion of unclaimed dogs rehomed (71%), compared to cats (45%). Telephone questionnaires were conducted with animal management officers from 35 councils (44%). Those with low euthanasia rates had high reclaim rates and/or rehome rates. Reclaim, rehome and euthanasia rates for dogs and cats were not significantly different between councils that operated their own pound facilities and those that utilized the services of welfare organizations to operate pounds on behalf of the council. More council managers believed they would never achieve ≤10% euthanasia for cats (49%) than for dogs (11%). A variety of strategies were used by councils to achieve high reclaim and rehoming rates.

## 1. Introduction

In developed countries, euthanasia of dogs and cats in animal shelters is the primary cause of death for healthy and treatable companion animals [1]. Current estimates by the American Society for the Prevention of Cruelty to Animals (ASPCA) are that approximately 6.5 million dogs and cats enter shelters across the United States of America (USA) each year, and that 1.5 million are euthanized [2]. In the year 2016–2017, the Royal Society for the Prevention of Cruelty to Animals (RSPCA) in Australia reported that 44,770 dogs and 53,912 cats entered their shelters nationally; and of these, 13% of dogs and 27% of cats were euthanized [3].

Euthanasia of healthy and treatable animals imposes a significant cost, affecting the health and well-being of the shelter staff involved [4], and has financial, moral, ethical and emotional ramifications for the community [5,6,7,8]. Many workers involved in euthanasia develop perpetration-induced traumatic stress [4,8], which increases the risk of addictions and suicide, and leads to increased sick leave and staff turnover. This results in increased rehiring and retraining costs for the shelter [7], and fosters a negative community attitude towards shelters and pounds [9].

Addressing concerns surrounding euthanasia of stray and surrendered dogs and cats in shelters involves finding alternative strategies to decrease intake and increase the number released alive, either through reclaim by their owners, adoption, or transfer to a rescue group or other agency for adoption. In the USA, attempts to reduce euthanasia have focused on reducing the number of animals entering animal shelters, as this is associated with improved outcomes for animals [10]. For example, subsidized spay/neuter (sterilization) programs targeted to locations of high shelter intake have been shown to be effective in decreasing cat intake and euthanasia, although this had less of an impact on reducing dog intake and euthanasia [11,12,13]. Targeted trap–neuter–return programs also assist in controlling urban stray cat populations, and reduce intake and subsequent euthanasia in shelters and council pounds [14,15,16].

In Australia, domestic animal welfare and management is regulated by state legislation. In the state of Victoria, legislation requires dogs and cats over three months of age to be micro-chipped, and the owner’s contact details registered with a nationally recognized database. Animals must also be annually registered (licensed) with the council (local government authority similar to a county in the USA) where the animal resides. Under the Victorian Domestic Animals Act 1994, councils have discretionary power to implement local laws to set dog and cat registration fees, and set fines for breaches of their local laws in relation to dog and cat management. Some councils require dogs and/or cats to be sterilized, and some councils have night-time cat curfews. Councils set limits on the number of each species of animal that can be registered at an address, and can ban pet ownership from environmentally sensitive areas. If dogs and cats are found wandering at large outside their property, the owner is liable for a financial penalty designated by the council. Councils also have the power to impose financial penalties on those who harbor unregistered (unlicensed) pets at their residence, or are in possession of unregistered dogs or cats, although compliance may be modest, and lower for cats than dogs [17].

Enforcement of state legislation and council by-laws is the responsibility of local officials in each council area, who may also have other local duties such as parking control. Such local officials, commonly known as animal management officers, frequently deal with compliance issues and educate owners about responsible pet ownership. In addition, they investigate and deal with animal complaints from the public on issues such as dog attacks, animals at large, strays on property and barking dogs.

Councils are also involved in managing lost and unwanted dogs and cats. Under Victorian legislation, councils must hold unidentified stray dogs and cats for a minimum of eight days, during which owners can reclaim their animal. However, after this time the animal becomes the property of the council, which can then choose to rehome the animal, transfer it to a welfare organization or rescue group, or euthanize it. Many councils choose not to operate their own impound facilities, instead outsourcing this to animal welfare agencies. In these situations, some councils provide physical facilities, and welfare agency staff manage the operation, while others transfer animals immediately (or within 48 h) to off-site facilities owned and operated by welfare organizations. A minority of councils perform all processes from admission to discharge or euthanasia on their premises, and are commonly called a council pound. Although impounded animals legally remain the council’s responsibility for a minimum of eight days, some contract welfare organizations to attempt reunification of animals with their owners, rather than allocate internal resources to this endeavor.

The Victorian Government has implemented several initiatives to promote and maintain animal welfare. For example, councils have discretion to offer alternatives to convictions and fines for breaches of the Act. For dogs, this can include undertaking compulsory training in responsible dog ownership, known as the Responsible Dog Ownership Course. The course is administered online, and aims to educate dog owners about their rights and responsibilities, as well as their dog’s welfare requirements. Other initiatives recently introduced include legislation to prevent puppy farms, and restrict the sale of dogs and cats in pet shops to those sourced from registered pounds, animal shelters or foster carers (although this legislation was not in place at the time the survey was conducted). A small portion of annual registration fees collected by councils is allocated to the state government to pay for the promotion of responsible dog or cat ownership and animal welfare, research into domestic animals management and the administration of the Domestic Animals Act 1994.

In addition to these initiatives, Victorian councils are required (under Section 68A of the Domestic Animals Act 1994) to prepare a Domestic Animal Management (DAM) Plan every four years. This plan must outline programs, services and strategies the council intends to pursue to address overpopulation and high euthanasia rates for dogs and cats within its municipality [18]. Although not mandatory, most Victorian councils report intake and outcome statistics for dogs and cats originating from their council area, providing a valuable database to analyze. Based on intake and outcome data from the 2013–2017 DAM Plans (data not shown), some councils were achieving euthanasia rates for dogs ≤10%, while others were euthanizing 45–55% of dogs and over 90% of cats. Although obvious factors which impact euthanasia are the proportion of animals reclaimed by owners, and the proportion found new homes (adopted), the number of animals admitted (intake) also impacts on the proportion euthanized [10]. Currently, there are no reports analyzing the publicly-available Victorian DAM Plan data, to assist in identifying strategies employed by councils that result in reduced admissions and euthanasia, and increased reclaim and rehoming rates.

The aims of this study were, firstly, to analyze data from the current 2017–2021 DAM Plans of all 79 Victorian councils with available data; and secondly, to conduct a survey to examine policies, practices and attitudes of council staff. It is anticipated that by benchmarking performance relating to intake and outcomes, and by identifying innovative strategies used by councils, this may serve as a guide for councils and welfare agencies regarding effective use of resources to reduce animal admissions, and improve expected outcomes for all dogs and cats admitted.

## 2. Materials and Methods

This two-part study was approved by the University of Queensland Animal Ethics Committee (#2015000738). In the first part of the study, data relating to dog and cat admissions, as well as numbers reclaimed, rehomed, and euthanized, were recorded from the DAM Plan of each council for the year 2016–2017 (or if unavailable, the year 2015–2016). Those with no available DAM Plan data for the relevant years were contacted via telephone or email to provide the relevant statistics. The number of dogs and cats registered (licensed) and registration costs were also obtained for each council using website information. Socio-economic status on a 1–10 scale was obtained for each council from the Know-Your-Council website [19]. For calculation of the scores, the Victorian Government inputs data from various council data annual reports and uses the Index of Relative Socio-Economic Disadvantage which summarizes 17 different measures, such as income, education, unemployment and unskilled occupations [20].

Councils across Victoria face a variety of factors that influence their ability to manage dogs and cats in their respective regions. Given the variation between councils in metropolitan and regional areas, council classifications were used to examine whether demographical factors contributed to the results. The classifications used were based on the Australian Classification of Local Governments [21]. Local government areas (councils) in all Australian states are classified according to their population, population density and proportion of rural and urban populations. Urban areas are classified as capital cities, metropolitan developed, regional towns/cities, or fringes; and rural areas are classified as agricultural, remote or significant growth areas. For the analyses, the capital city (Melbourne) was included in the urban metropolitan development category, and all rural subcategories were grouped into a single rural category.

In our study, we also report the proportion of unclaimed dogs adopted as it is a more meaningful measure of adoption activities, because of the influence of return to owner on the proportion of intake adopted. Efforts to rehome animals are masked when reclaim rates are high, and if highly variable between facilities, using proportion of intake adopted precludes meaningful comparison of adoption efficacies between sites.

The second part of the study consisted of a survey of council animal management staff about policies, practices and beliefs, to identify strategies used to decrease dog and cat admissions and euthanasia in council pounds. A survey was undertaken in 2015–2016 of councils across Victoria. The survey was divided into 16 sections comprising questions on the following topics: animal management and processing practices; promotion of responsible pet ownership; attitudes towards rehoming all treatable and adoptable dogs and cats; feral and stray cat management; and attitudes towards trap–neuter–return programs. During the questionnaire development, a pilot study was performed at a local government facility in Queensland, where the survey design was refined and language clarified. The questionnaire was modified accordingly.

From 1 May 2015 to 30 September 2015, all 79 councils were contacted to invite their participation in a telephone questionnaire. Most councils were contacted by telephone, but due to difficulty contacting key staff and time constraints, some were emailed. A person was identified from each council with an appropriate level of knowledge regarding local pound protocol and operations. A copy of the survey was sent to each council which agreed to participate, and a time was established to conduct the survey. Telephone questionnaires were completed by three investigators (GI, LM, CC). Responses were recorded in writing at the time of the interview, and entered in an electronic spreadsheet. Participation was voluntary, and participants were informed that their non-publically available data would be de-identified when reported, unless the council expressly gave permission for the data to be published. Most questionnaires were completed by phone between May 2015 and December 2016, with a few completed by email. An overall participation rate of 44% (35/79) was achieved.

Participants were informed that overseas there had been some success in controlling unowned cat populations. They were provided with the URL of a paper describing the outcome of such a program in San Jose, USA [12]. In that study, cat intake into shelters was reduced in programs where stray cats were sterilized and then returned to the location where they were found. Participants were then asked about their attitudes towards a trap–neuter–return (TNR) program, and if they thought it could be effective in urban areas of Australia.

Descriptive statistics reported in all tables include means, standard deviations, medians, quartiles, percentages and proportions; with medians presented in text for all intake and outcome statistics, given the skewed nature of the data. Statistics for total intake represent all dogs and cats admitted to councils, regardless of outcome. The proportion of each outcome (i.e., reclaimed, rehomed and euthanized) were calculated using an adjusted intake that excluded the following animals: Stolen/escaped; unassisted deaths/deceased on entry; and animals being processed/held or fostered at time of statistics publication and therefore would be accounted for in the following year of data).

Due to the unequal sample sizes and variances between demographic and facility groups, the non-parametric Kruskal–Wallis one-way analysis of variance (ANOVA) for independent samples was used to analyze all population, registration, intake and outcome data. The significance values were adjusted by the Bonferroni correction for multiple tests. Step-down follow-up analyses were undertaken for significant results to identify where the differences were. Independent *t*-tests were used to compare registration prices for entire and sterile dogs and cats in high and low socio-economic areas. The non-parametric statistic Spearman’s correlation coefficient was used to test whether the proportion of dogs and cats euthanized was associated with animal intake per 1000 residents; and if the proportion reclaimed or proportion of unclaimed animals rehomed were associated with the proportion euthanized. Chi-square analysis was used to evaluate the relationship between attitudes of councils towards euthanasia and rehoming rates (above or below state average). Analyses were performed using IBM SPSS Statistics for Windows (version 25, 2017); and for all analyses, the significance level was *p* ≤ 0.05. Methods used to increase reclaim and rehoming in our survey cohort are descriptively reported, because only six of the 35 councils surveyed were full-service operations that operated their own pound facilities and managed their rehoming programs.

## 3. Results

### 3.1. Demographic Information for the State and Survey Cohort

Overall, human populations in the council areas ranged from 2904–313,521 (Table 1). Urban metropolitan developed and fringe areas had the largest human populations and were, on average, more than 10 times larger than rural agricultural populations (Table A1). These areas also had the highest total number of dogs and cats registered. Mean number of animals registered was 150 dogs/1000 residents and 43 cats/1000 residents. Socio-economic status of councils varied from a low of 1 to a high of 10 across the state, with a mean of 5.6; and urban regional and rural agricultural councils had a significantly lower socio-economic status than urban metropolitan developed and fringe areas (H(3) = 27.971, *p* < 0.001).

Results of independent *t*-tests showed the price of registration for entire dogs was significantly higher for those in high socio-economic areas (6–10 on the 10 point scale) (M = $144.49, SD = $39.88) compared with lower socio-economic areas (1–5 on the 10 point scale) (M = $120.69, SD = $29.83) (*t*(76) = 2.959, *p* = 0.004). For sterilized dogs, registration was also significantly higher for those in high socio-economic areas (M = $44.92, SD = $10.48) compared with lower socio-economic areas (M = $38.48, SD = $8.13) (*t*(77) = 3.017, *p* = 0.003). There was no significant difference in mean price for an entire cat for those in high or low socio-economic areas (M = $116.33, SD = $32.02 versus M = $107.30, SD = $32.13) (*t*(68) = 1.178, *p* = 0.243); or for a sterile cat (M = $35.03, SD = $9.40 versus M = $34.85, SD = $9.65) (*t*(77) = 0.084, *p* = 0.933). While all councils offered pensioner concessions of 50% for registrations, this did not include Health Care Card holders, who are low income earners. Three out of the 35 councils surveyed were noted as requiring all newly registered dogs and cats to be sterilized, and a further 14 had this requirement for all newly registered cats.

### 3.2. Categorization of Participating Councils by Type of Animal Management Operation

Over half (66%) of the 35 councils in our survey cohort outsourced dog and cat care to an animal welfare agency immediately, or within 48 h of admission (immediate transfer); 17% ran the entire pound operation from admission to discharge/euthanasia (full-service); 11% held dogs and cats at the council facilities for the eight-day mandatory holding period and then transferred them to an animal welfare agency (hold); and 6% held dogs for the eight-day holding period but immediately transferred cats to an animal welfare agency (Table 2). In all demographic areas, immediate transfer operations were the most common service type.

Of those councils transferring to welfare organizations (83%, 29/35), either immediately or after the eight-day holding period, most were to the Lost Dogs Home (31%, 9/29), RSPCA (28%, 8/29) and Animal Aid (14%, 4/29). Some of the other organizations included: Blue Cross; Maneki Neko Cat Rescue; Cat Protection Society; Geelong Animal Welfare Society; Pets Haven; and local veterinarians. Some councils that operated their own pounds also transferred animals to welfare organizations on an ad hoc basis for adoption. Victorian legislation stipulates that animals must be sterilized, vaccinated and micro-chipped prior to adoption [24]. Aside from meeting these legislative requirements, only 15% (4/27) of these councils reported having key performance indicators (KPI’s) incorporated into the contract regarding rehoming or euthanasia targets for animals.

### 3.3. Intake and Outcome Data for Impounded Dogs and Cats

#### 3.3.1. Dog and Cat Intake

Based on DAM Plan data, median dog admissions for the state were 6.5 dogs/1000 residents, but ranged widely for individual councils from 0.7 to 24.6/1000 residents (Table 3). For cats, median intake was 6.5 cats/1000 residents, also ranging widely between councils from 0 to 27.6 cats/1000 residents. The highest quartile had intake rates of 10.4 or more dogs and 10.5 or more cats/1000 residents. For the survey cohort, median admissions were similar to the state average for dogs (6.6 versus 6.5 dogs/1000 residents), but lower for cats (5.7 versus 6.5 cats/1000 residents). Urban regional and rural agricultural areas had significantly higher median intakes than urban metropolitan developed and fringe areas of both dogs (10.9 and 7.2 versus 2.5 and 4.9 dogs/1000 residents respectively [H(3) = 43.170, *p* ≤ 0.001]) and cats (10.1 and 9.5 versus 1.9 and 4.3 cats/1000 residents respectively [H(3) = 34.574, *p* ≤ 0.001]) (Table A2). Based on the survey data, there was no significant difference in dog and cat intake between operation types (Table 4).

#### 3.3.2. Dog and Cat Outcome: Reclaimed

The median proportion of dog admissions reclaimed by their owners was similar for both state and survey cohorts (77% and 81% respectively). The highest quartile for the state had 87–100% of admitted dogs reclaimed, and the lowest quartile of councils had 33–61% of dogs reclaimed. The proportion of dogs reclaimed varied between demographic groups, with significantly more dogs reclaimed from urban metropolitan developed and fringe areas (H(3) = 30.883, *p* < 0.001). Based on the survey data, there was no significant difference in reclaim rates for dogs between operation types.

Median reclaim proportions for cats in the state and survey cohorts were 10% and 13% respectively. The highest quartile for the state had 17–59% of cats reclaimed, and the lowest quartile had 0–5% of cats reclaimed. For cats, reclaim rates varied from 0–59%, and were significantly different between demographic areas (H(3) = 20.028, *p* < 0.001), but not between operation types. 

#### 3.3.3. Dog and Cat Outcome: Rehomed

The median percentage of dogs admitted that were rehomed was 16% for the state and survey cohorts; and the percentage of unclaimed dogs rehomed was similar, being 72% and 75% respectively. The highest quartile for the state rehomed 84–100% of unclaimed dogs, and the lowest quartile rehomed 21–63% of unclaimed dogs. A significantly higher proportion of dogs were rehomed in urban regional and rural agricultural areas compared with urban metropolitan developed and fringe areas, reflecting the lower proportion reclaimed (H(3) = 25.670, *p* < 0.001), but this was not significant for the proportion of unclaimed dogs rehomed. Median percentage of total intake rehomed, and of unclaimed dogs rehomed, was not significantly different between operation types.

The percentage of cats rehomed was lower for the state (39%) than survey cohort (52%), as was the percentage of unclaimed cats rehomed (49% versus 58%). The highest quartile for the state rehomed 59–81% of unclaimed cats, and the lowest quartile rehomed 0–19% of unclaimed cats. There were no significant differences in the proportion of cat intake rehomed or proportion of unclaimed cats rehomed between demographic groups or operation types, but there was a large variation within each category.

#### 3.3.4. Dog and Cat Outcome: Euthanasia

The median percentage of dog admissions that were euthanized for the state and survey cohorts was 6% and 5% respectively. Most (74%; 52/70) Victorian councils achieved a euthanasia rate for dogs of ≤10% of intake, often considered as representing zero euthanasia of all healthy and treatable animals. The highest quartile for the state euthanized 11–40% of dogs, and the lowest quartile euthanized 0–3% of dogs. The proportion of dogs euthanized was significantly different between demographic areas (H(3) = 17.947, *p* ≤ 0.001), but not operation types.

The median percentage of cat admissions that were euthanized for the state was 43% and the survey cohort was 34%. Only one council (Maroondah) achieved ≤10% euthanasia of cat admissions, with 6.5% euthanized from 1.3 cat admissions per 1000 residents (immediate transfer to Animal Aid, intake 155, 26% reclaimed, 91% unclaimed cats rehomed). The highest quartile for the state euthanized 67–98% of cats, and the lowest quartile euthanized 7–28% of cats. The proportion of cats euthanized was not significantly different between demographic areas or operation types.

Spearman’s correlation coefficient analyses showed euthanasia for dogs was not correlated with intake per 1000 residents (*r*(70) = 0.073, *p* = 0.547), but this was significant for cats (*r*(69) = 0.282, *p* = 0.019). Percentage euthanized was negatively associated with the proportion reclaimed for dogs (*r*(70) = 0.699, *p* ≤ 0.001) and cats (*r*(69) = 0.402, *p* = 0.001), as well as the proportion of unclaimed dogs rehomed (*r*(69) = 0.669, *p* ≤ 0.001) and unclaimed cats rehomed (*r*(69) = 0.961, *p* ≤ 0.001).

### 3.4. Council Protocols for the Euthanasia of Impounded Dogs and Cats

All surveyed councils were asked about final decisions for feral cat classification, and subsequent euthanasia, and who they were made by (Table 5). For full-service and hold operations, few (17% and 26%) sought the advice of veterinarians to assess whether cats were feral, instead allowed council rangers or other staff to classify these cats. Most immediate transfer operations (74%) left the final assessment regarding feral cats and subsequent euthanasia to the transferring shelter, and it was unknown if they were assessed by veterinarians or other shelter staff.

Full-service operations were asked about their procedures regarding euthanasia and the frequency of euthanasia for animals under different conditions (Table 6). Most (83%, 5/6) full-service operations had defined criteria for dog euthanasia, but fewer (50%, 3/6) had defined criteria for cat euthanasia. One full-service facility reported always euthanizing kittens with their mother if the mother was classified as feral based on behavior and lack of identification.

### 3.5. Strategies Used by Councils to Reduce Euthanasia

The primary strategies used by councils to reduce euthanasia of impounded dogs and cats can be divided into three major categories: (1) Reducing animal intake; (2) increasing animal reclaim rates; and (3) increasing rehoming rates.

#### 3.5.1. Strategy 1: Reducing Animal Intake

Our survey cohort used multiple strategies to reduce the intake of dogs and cats, including: (1) Promoting responsible pet ownership; (2) returning roaming animals directly to owners; (3) escalating fines for owners of animals caught repeatedly wandering; (4) subsidized sterilization programs to reduce unwanted puppies and kittens; (5) offering alternatives to surrender; and (6) slowing intake when at or near capacity (Table 7). Under the Victorian Government Domestic Animals Act 1994, all councils are required to promote responsible pet ownership behaviors and they used a variety of methods.

*Returning roaming animals directly to owners:* Although under the Domestic Animals Act 1994 of Victoria, roaming animals must be impounded, nearly all of the survey cohort directly returned roaming animals with current registration (licensing) tag and microchip to their owner, if contactable. A few councils returned identifiable but unregistered animals, and had owners complete the registration paper work at the point of return or later, and followed up for payment. More councils did this at times of adverse weather conditions or firework activity and when nearing capacity. Many councils (89%) allowed officers to use their discretion regarding whether they would issue a warning or fine for roaming animals, and nearly all councils (97%) levied escalating penalties for repeat offenders. One council, with an intake of 6.3 dogs/1000 residents and high reclaim (86%) for dogs and cats (17%), utilized a 24-h seven-days-a-week hotline for owners to report missing animals. This information was immediately sent to all staff whether they were on duty or not, so they could look out for the animal and return it home.

*Subsidized sterilization programs:* Only 40% of councils provided subsidized sterilization programs, and these were either operated by the council directly, or in conjunction with rescue groups. Of those with subsidized programs (*n* = 14), five were in the form of Australian Veterinary Association (AVA)/Municipal Association of Victoria discounts for people on limited income whereby councils subsidized 33% of the cost and the owners paid the remaining 67%. Three programs were conducted in collaboration with rescue groups but were on an ad hoc basis. Two councils restricted programs to pensioners and concession card holders. Only 63% of respondents were aware of the availability of AVA vouchers for subsidized sterilization, and of those aware, only 53% distributed them. One council that utilized them commented that the process was unduly time consuming. Other councils who were aware but not using them commented that council contributions to sterilizations made them unaffordable to the council, especially if they felt the participating veterinary practices were not also subsidizing the costs.

One council reported a 25% reduction in wandering dogs in the two years following the council introduction of mandatory sterilization of dogs (despite an initial increase in numbers). Another council, however, was uncertain whether there were any benefits to the introduction of compulsory sterilization of dogs, noting that some people wanting to reclaim their pets from the pound could not afford the required sterilization fees. One council offered free sterilization clinics for cats twice a year. Numbers were limited to approximately 65 cats per clinic with a budget of $15,000 per annum to sterilize and microchip cats. To increase compliance for owners of queens that had produced previous litters of kittens, animal management officers transported the queens for sterilization, because this was identified as a barrier to seeking veterinary services. In the four years following introduction of this initiative, the council observed a marked decline in the number of litters surrendered. The council also used AVA vouchers, and offered free sterilization for potential animal welfare situations, such as dogs that previously had several litters. The number of dogs impounded decreased from 363 in 2012 to 191 in 2016, while cat intake decreased from 740 to 218 in the same period.

*Offering alternatives to surrender:* For owners surrendering animals, all responding councils recorded and/or discussed reasons for the surrender with the owner; and over half (57%) utilized strategies to encourage owners to keep their animal and offered solutions.

*Slowing intake when at or near capacity:* When facilities were nearing capacity, 27% councils attempted to slow the number of incoming dogs and cats using various strategies including reducing the number of cat traps available.

#### 3.5.2. Strategy 2: Increasing Animal Reclaim Rates

The main strategies used by councils to increase the reclaim of dogs and cats by their owners were: (1) Increasing animal identification through registration compliance monitoring and subsidized micro-chipping events; (2) advertising stray animals; and (3) extended holding and reclaim times for identified animals (Table 8).

*Increasing animal identification:* Almost all (97%) councils conducted compliance monitoring to increase current registrations. Of these, all conducted door knocking, with the knocking typically targeted to those with lapsed registration, rather than general door knocking to identify animals that were never registered. Some utilized microchip registry data to extract the details of owners of animals living in postcodes within that council, and identify those without current registration. However, only 17% ran micro-chipping events to increase the number of animals with microchips, and the accuracy of the owner contact details. One council targeted these to young and older pets.

*Advertising stray animals*: Most councils that advertised stray animals used their council website, while a minority used Facebook, local newspapers, rescue group websites, and one posted brochures at veterinary clinics and on a community app.

*Extended holding and reclaim times*: One immediate transfer council that achieved very high reclaims for dogs and cats (92% and 26%, respectively) held identified stray animals until the evening at the council offices and attempted to contact owners, so the animal could be delivered to, or picked up by the owner, rather than transferred to the welfare agency. Owners could pick up animals over weekends and until 8:30 pm on weekdays. Of animals reclaimed, 77% were reclaimed from the council offices without being transferred. Those that were not registered could fill out the paperwork at the time of picking up the animal, or when it was delivered home.

#### 3.5.3. Strategy 3: Increasing Rehoming Rates

All surveyed councils were asked about: (1) Their vaccination protocols to reduce illness and increase adoptability of animals, and (2) whether they encouraged those admitting stray animals to adopt them (Table 9). Full-service facilities were also questioned about their adoption, volunteer, fostering, rehabilitation and transfer programs.

*Vaccination protocols:* Only one full-service operation routinely vaccinated dogs and cats, and this was not on entry, but within 48 h depending on the veterinarian’s ability to attend the shelter. One hold operation vaccinated dogs for parvovirus on entry. All full-service (*n* = 6) and hold (*n* = 6) operations reported undertaking some routine medical treatments when required.

*Encourage finders of stray animals to adopt:* Many respondents (70%) across full-service, hold and immediate transfer operations encouraged those bringing in/reporting strays to adopt them if unclaimed, and either recorded their expressions of interest on the relevant paperwork, or directed them to the shelter they were transferred to.

*Programs in full-service facilities:* All (*n* = 6) full-service operations had adoption programs, whereby animals were directly adopted out to the public from the council pound. Various strategies were used to facilitate adoptions. One full-service operation had a special induction/education building with one room set up in a home-like environment with a couch, so potential adopters could bring their family and pets to spend time with council dogs or cats they were considering adopting. Two full-service operations had implemented, and two were in the process of developing volunteer programs whereby volunteers walked, groomed and feed animals, and assessed their suitability for adoption. Half (50%, 3/6) of the full-service operations utilized fostering programs, which were provided by members of local rescue groups and veterinary clinics. When asked how effective councils thought the fostering program was at increasing the rehoming or transfer rates of animals, all three respondents believed it was “very” to “extremely” effective. Their percentage of unclaimed dogs rehomed ranged from 72–88% and for cats ranged from 55–74%, respectively.

Only one full-service operation had a formal rehabilitation program to improve the adoptability of animals. For dogs, this included providing basic training and improving socialization, especially for timid animals. This council also provided similar socialization programs for cats, with timid cats given more time to adapt, or removed from the shelter environment by a rescue group. When asked how successful they believed the rehabilitation program was, the respondent stated that it was “extremely” successful for both dogs and cats. The percentage of unclaimed dogs and cats adopted were 88% and 74%, respectively.

Almost all (83%, 5/6) full-service operations worked in collaboration with rescue organizations, however only one of these councils utilized adoptions through pet shops as well. Those full-service operations that did collaborate with rescue organizations were asked several questions regarding the reasons for transfer, and most commonly were transferred to increase their chance of adoption (Table 10).

### 3.6. Attitudes towards Zero Euthanasia of Treatable and Adoptable Dogs and Cats

Animal management officers were asked a series of questions relating to euthanasia and their attitudes towards achieving zero euthanasia of treatable and adoptable dogs and cats (Table 11). When asked whether it would be possible to rehome all treatable and adoptable animals admitted to their shelter, given sufficient resources, Chi-square analysis showed a significantly higher proportion of respondents believed that their council could achieve this for dogs (86%) than cats (40%) (*X*^2^ (1, *N* = 70) = 15.664, *p* ≤ 0.001). For dogs, the respondents’ beliefs in respect of their ability to rehome all treatable and adoptable dogs was not correlated with whether they rehomed more unclaimed dogs than the state average of 71% (*X*^2^ (1, *N* = 32) = 0.463, *p* = 0.496). However, this was significant for cats (*X*^2^ (1, *N* = 30) = 6.531, *p* = 0.011), indicating that respondents with a positive attitude towards rehoming all treatable and adoptable cats were more likely to be rehoming greater than the state average of 45% of unclaimed cats. Most officers from full-service facilities, hold and immediate transfer operations believed all treatable and adoptable dogs could be rehomed (100% and both 83% respectively).

When asked how many years it would take their council to achieve zero euthanasia of all treatable and adoptable dogs if the required resources were available, 66% (23/35) said they were already achieving this or could achieve this target in less than 10 years, while 17% (6/35) said never. For cats, 40% (14/35) said they were already achieving this or could achieve this target in less than 10 years, and 29% (10/35) said never. Three of the six full-service operations were of the belief they already rehomed all treatable and adoptable dogs (euthanasia rates 2%), and two believed they also rehomed all treatable and adoptable cats (euthanasia rates 19–22%).

Participants were then asked what barriers prevented them rehoming all treatable and adoptable dogs, with almost half of the respondents (40%, 14/35) stating that they would require increased funding and resources (staff, facilities, and foster carers). Other barriers included the cost of animal ownership (*n* = 3), too many dogs (*n* = 3), placing animals with behavioral problems with appropriate people (*n* = 2), and adoption costs (*n* = 1). When asked about the barriers to rehoming all treatable and adoptable cats, funding and resources was also the most common barrier (26%, 9/35), followed by too many cats to be rehomed (23%, 8/35) and a lack of pet ownership education (14%, 5/35), which contributed to unsterilized animals and too many cats in the community. Other barriers included legislation (*n* = 3), cat health issues (*n* = 2), lack of community support (*n* = 2), and the cost of animal ownership (*n* = 1).

Regarding where council should focus efforts to see the greatest decrease in euthanasia for dogs, 49% (17/35) felt that educating owners about responsible pet ownership would be productive, and 37% (13/35) of respondents believed that increased access to sterilization programs would result in the greatest reduction for dogs. The remaining responses suggested improved micro-chipping and identification rates (*n* = 3) and ensuring that dogs were matched to suit the owner’s lifestyle (*n* = 2). Another mentioned that undertaking research that focused upon the demographics and socio-economics of areas with high euthanasia could inform future strategies.

For cats, 51% (18/35) would focus on sterilization to decrease euthanasia of cats. A further 34% (12/35) would target the promotion and education concerning responsible cat ownership. Other responses included increasing cat identification methods (micro-chipping and registration) (*n* = 2), amending current legislation (*n* = 2), and further research regarding the high euthanasia rates in cats (*n* = 1). One respondent felt that to achieve zero euthanasia of all suitable cats, it was necessary to change the Australian public’s views on cats as being ‘disposable items’. Another mentioned a current lack of community response and support, in the sense there was not enough community concern about the welfare of cats admitted to the pound that would drive efforts to address this issue.

When managers were asked about their attitude towards transferring animals to pet shops for rehoming, they responded with openness (59%, 13/22), opposition (23%, 5/22), and mixed feelings (18%, 4/22) towards the concept. Reasons for opposition included risks of impulse buying of animals, and taking business away from the RSPCA. Of the five full-service operations that regularly transferred animals to other welfare organizations, four felt positively towards transferring animals to pet shops to increase the opportunity of adoption, and two were already doing so.

### 3.7. Cat Trapping and Council Attitudes towards Trap–Neuter–Return Programs

Many (57%, 20/35) councils undertook some trapping of unowned cats, although half of these only did so in response to complaints from the public. All councils had traps available for the public to use. Of those councils that could provide details regarding the number of cats trapped in the previous year (*n* = 13), this ranged from 4 to over 1200 unowned cats.

In response to the question about their attitudes towards a trap–neuter–return (TNR) program, and if in their opinion, it would be effective in urban areas of Australia, over half of respondents 54% (19/35) did not believe it would be effective, 40% (14/35) believed it would be effective and 6% (2/35) were unsure of their views on this topic. Most respondents (77%, 27/35) were concerned about the impact of cats on wildlife if a TNR program was implemented, even if the cats were neutered. A quarter of the respondents (26%, 9/35) were concerned about the cost of running such a program; 29% (10/35) noted the public’s opposition to such a program as a barrier to implementation; and 37% (13/35) were concerned about the welfare of the cats included in the TNR program.

One council currently enforced a 24-h on-property cat curfew, and voiced concerns that implementing a TNR program would provide a confusing message to cat owners in their council. Regarding funding, respondents suggested this could be obtained from the state government, private organizations, or included as part of council animal registration fee. Respondents also suggested that veterinarians could support such a program by providing discounted services, or enabling student veterinarians to assist in these programs (under supervision) to obtain clinical experience.

## 4. Discussion

This study analyzed all available intake and outcome statistics for dog and cat admissions to the 79 councils in Victoria, and there were several noteworthy findings. Based on self-reported data in the Domestic Animal Management Plan for each council (or obtained via their website or personal communication), 74% (52/70) of Victorian councils have achieved a euthanasia rate for dogs of ≤10% of intake. This is generally accepted as equivalent to achieving zero euthanasia of healthy and treatable animals [25]. Notably, the best performing 25% (quartile) of councils have bettered this, with euthanasia rates of 0–3%, while the poorest performing quartile euthanized 11–40% of dogs. Only one council achieved a euthanasia rate of ≤10% of admissions for cats. For cats, the lowest quartile euthanized 7–28%, while the upper quartile euthanized 67–98%. For both dogs and cats, there were no differences in outcomes between councils that operated their entire pound operation or those transferring to welfare agencies, either immediately, or after the minimum holding period, which in Victoria is eight days, except for abandoned animals (14 days for animals illegally and intentionally abandoned). In general, rural and regional areas had higher euthanasia than urban metropolitan developed and fringe areas.

In this study, we also surveyed practices, policies and attitudes of animal management staff at 35 councils to identify strategies used to decrease euthanasia. In our survey cohort, only 17% of respondents ran full-service facilities, while the other 83% outsourced dog and cat care by transferring them to an animal welfare agency either immediately, within 48 h of admission (66%), or after the eight-day mandatory holding period (17% for dogs). Some important findings associated with low euthanasia in the council pounds were identified, including strategies to reduce intake and achieve high reclaim and adoption rates of dogs and cats.

Only 22% of councils contracting welfare agencies to perform services incorporated Key Performance Indicators (KPI’s) into the contract; and of these, only two incorporated KPIs relating to the agency’s performance regarding proportion euthanized (dog euthanasia: 11–18% and cat euthanasia: 27–51%). Councils utilizing KPI’s in contracts with animal welfare agencies have the potential to achieve better outcomes for their community animals, particularly cats. Community pressure to achieve zero euthanasia of healthy and treatable animals is affecting how welfare organizations interact with council agencies, resulting in fewer agencies being prepared to accept council contracts, or charging higher rates per animal to reflect the total cost of caring for animals until they are adopted [26].

### 4.1. Strategies Used by Councils to Reduce Euthanasia: Reducing Animal Intake

In our study, intake per 1000 residents for the state varied from 0.7–24.6 for dogs and 0–27.6 for cats. The only council that achieved <10% euthanasia of cats had an intake of 1.3 cats/1000 residents. For cats but not dogs, intake was negatively associated with euthanasia (*p* = 0.009). In dogs, the lack of effect is likely because most councils directly returning lost animals (mainly dogs) to their owners, included these animals in the number managed (intake), although they did not enter their shelter/pound. Reducing dog and cat admissions into pounds is pivotal in reducing euthanasia rates. Over the past 30 years in USA, the decline in euthanasia rates has been largely attributed to a decline in shelter intake for both dogs and cats [10,27]. However, as euthanasia rates decrease, increasingly adoption becomes a substantial contributor to further reducing euthanasia [10]. Multiple strategies were used by our survey cohort to reduce intake, particularly for dogs.

#### 4.1.1. Reducing Intake by Minimizing the Number of Surrendered Animals

Research shows that most owners surrendering pets would keep them if the barriers to retention were resolved [28,29]. Barriers to retaining pets include: cost for veterinary care; pet registration (licensing) or impound fees; behavioral problems; and a lack of pet friendly accommodation. Pet retention programs which address these issues substantially reduce intake.

Many councils had strategies in place to assist owners to avoid surrendering an animal to minimize intake, especially when nearing capacity (57%). These included temporarily housing a dog while means of confinement were repaired, or providing information on animal-trainer contacts for behavioral issues. Research shows that most people surrendering a pet are experiencing financial hardship and would keep their pet if the barriers were minimized. Studies from the USA show providing assistance with impound or registration fees (35%), pet care costs (27%) and fencing (15%) are the most common types of assistance required to help owners retain their pets [28,29]. RSPCA Queensland is an Australian example of the impact on cat intake of a diversion program. They provide resources on their website to assist potential surrendering owners with strategies and resources to keep their pet or rehome it themselves, and require all surrendering owners to have an appointment and then wait a further 14 days. This has helped halve the number of owner-surrendered cats over five years [30].

The full shelter costs for a dog or cat rehomed after one week are estimated to be $1056 and $756 respectively in Australia [31]. This includes admission costs, basic health care, sterilization, housing for one week, and other costs. When rehoming takes longer than one week, the shelter will incur additional costs of approximately $490 and $385 per week for dogs and cats respectively, to provide ongoing shelter care, including environmental enrichment. In comparison, it has been reported that the average cost of assisting owners to keep their pet was US$50 for assistance with fees (including reclaim or registration fees), US$150 for assistance with veterinary costs, and US$75 for assistance with fencing [28,29]. It is likely cost-effective for councils and shelters to invest resources to help people keep their pets.

#### 4.1.2. Reducing Intake through Mandatory Sterilization and Subsidized Sterilization Programs

Many respondents in our study believed that the most effective strategy to reduce intake and euthanasia was through sterilization programs and/or compulsory sterilization. However, only 40% of councils provided subsidized sterilization programs either in the form of a subsidy through the AVA program, vouchers, or in conjunction with animal welfare agencies. Most programs were only available to those on low income, and still required the owner to fund some of the cost; for example, 67% of the regular veterinary fee for the AVA program. Notably, Banyule City Council offered free sterilization clinics for cats twice a year and free transportation, mostly targeted to low socio-economic areas. Based on the resulting reduction in litter surrender, the council has since doubled the budget for this program. However, many respondents stated that funding was a major barrier to undertaking these subsidized sterilization programs.

Several studies demonstrate the effectiveness of sterilization programs for owned cats to decrease cat intake and euthanasia, and to a lesser extent, for owned dogs [32,33]. Data from the USA indicates that investing in these programs reduces the cost of animal management for councils, and that savings accrue from reduced husbandry and staff costs resulting from reduced admissions [34]. A 50% drop in cat shelter admissions occurred with a sterilization voucher program for cats, resulting in net savings to the county of $1.5 million [12,34]. For dogs, some studies support their effectiveness [12], and others show little effect unless targeted to areas associated with high intake [33]. A reduction in dog intake from 7.0 to 6.1 per 1000 residents occurred in the San Jose municipal pound with targeted free sterilization of Chihuahuas, the predominant breed being admitted [12].

Some councils had mandatory sterilization for newly registered cats and dogs, while others mentioned they were intending to implement this. However, a study of 191,000 cat admissions to RSPCA shelters in Australia from 2006–2010, demonstrated that the Australian Capital Territory had the second lowest sterilization rate for cats under 6 months of age, which was the only state or territory in Australia to have mandatory sterilization by 6 months at the time data were collected for the study [35]. This suggests that mandatory sterilization has limited effectiveness, and other factors are involved. In the USA, in the most underserviced and lowest socio-economic areas, sterilization rates are around 10% compared to the USA average of 90%. However, when free sterilization and transport are provided, sterilization rates approach 90% [36].

Based on this information, it is therefore recommended that councils trial free sterilization programs, targeted to locations with a high intake of dogs and/or cats, rather than relying on mandating sterilization to reduce intake. Although it could appear that councils that immediately transfer animals to welfare organizations might have less to gain from engaging in sterilization programs, reducing the number of animals transferred would potentially reduce the cost to council for third party services. It is recommended that welfare agencies consider charging councils the full costs for managing stray and surrendered dogs and cats, to encourage councils to invest more in programs in the community to decrease admissions of stray and surrendered animals, and their subsequent euthanasia.

### 4.2. Strategies Used by Councils to Reduce Euthanasia: Increasing Reclaim

Councils with higher reclaim rates had lower euthanasia for dogs and cats. Owned animals do stray, and in a 5-year period, 14% of dog owners and 15% of cat owners in the USA lost their pet at least once [37]. Even pets confined indoors are at risk, with 28–41% of lost cats described by their owners as “indoor only” [38,39].

If an unregistered animal is impounded, the owner may be liable to pay an impound release fee, sustenance fee, registration fee and a fine for failing to register their pet. In our study, areas with higher socio-economic status also had higher reclaims, which may indicate that finances may be important for reclaims. Penalties can be high and generally unexpected, meaning they cannot be budgeted for. Low-income earners may be unable to pay these fees, resulting in costs to council to rehome unclaimed animals. Although legally in Victoria roaming animals must be impounded, nearly all councils directly returned to the owner animals with current registration and a microchip, likely contributing to the very high reclaim rates. A few councils also returned unregistered animals if identifiable, and had owners complete the paperwork at the point of return, and followed up for payment later. Returning all animals where the owner could be contacted, even unregistered animals, is likely cost effective for councils, given the costs of impounding a dog if not reclaimed, and it is recommended that legislation is changed to allow this. Registration paperwork could be completed at the point of return, and payment processed in the field or followed up later. Providing evening and weekend times for contacting council offices regarding lost pets and extended pickup hours likely decreases the number of animals unclaimed, and it was utilized by one council (Glen Eira) with high reclaim rates for dogs (86%) and cats (17%).

Median reclaim rates for cats (10%) were lower than for dogs (77%). However, these rates were much higher than reported in a study from RSPCA-Queensland, where only 5% of cats and 37% of dogs were reclaimed [40], and an American Society for the Prevention and Cruelty to Animals study where <5% of cats and 26% of dogs were reclaimed [2]. The top quartile of Victorian pounds achieved very low euthanasia rates for dogs (0–3%), and most (66%) of these also had reclaim rates in the top quartile for the state (≥87% of dog intake). Many of the state’s pounds with the highest quartile for euthanasia (11–40%) were also in the lowest quartile for reclaim with 33–61% reclaimed. Based on these results, strategies used by councils that facilitate reclaim are important in determining positive outcomes for animals, and a variety of methods were utilized by the councils in our survey cohort.

#### 4.2.1. Increasing Reclaim through Identification of Animals with Microchips and Registration

Micro-chipping facilitates rapid identification of an animal, and reclaim rates are higher for micro-chipped animals than others (52% versus 22% for dogs, and 39% versus 2% for cats [41]. However, in a 2014 study, only 9% of stray cats and 28% of stray dogs entering RSPCA Queensland (QLD) were micro-chipped [42]. In 2009, Victoria was the first state to mandate micro-chipping for dogs and cats over 3 months, and the number of dogs and cats registered per 1000 residents on Australia’s largest data base, Central Animal Records, was greater than any other state [43]. Few councils (17%) engaged in community micro-chipping events, with one targeted at young and older pets.

Most (97%) councils undertook door-knocking for registration compliance, most of which was targeted at owners who had not renewed registration, rather than comprehensive door-knocking to detect animals that were never registered. Some councils utilized SMS reminders, telephone and email to advise or enquire about an un-renewed registration before door knocking. While door-knocking, some also offered to check microchip details when they were door-knocking for compliance checks.

Registration costs varied substantially between councils, from $10 to $285 per year, depending on whether the dog or cat was sterilized. Several councils were noted to only accept new dog and/or cat registrations if the animal was sterilized, and others were in the process of implementing this change. Research is needed to determine whether this policy represents a barrier to registration, and reduces reclaim of lost pets. While all councils offer pensioner concessions of 50%, this does not include Health Care Card holders, who are low income earners. Cost benefit studies are required to determine if it is more cost-effective to reduce registration costs to increase numbers of animals identified, than to gain income from registrations and pay higher costs for impounding more unidentified animals, which are likely held longer. Return on investment of identification programs should be investigated. It is also recommended a trial be conducted to evaluate the cost-benefit during door-knocking campaigns to check owners’ contact details on the microchip database. This could also involve evaluation of the merits of providing free identification tags engraved in the field with the owner’s contact details, and if necessary, a collar, so neighbors can assist in return of wandering pets directly to their owner, reducing unclaimed impounded animals and council costs.

#### 4.2.2. Factors Affecting the Reclaim Rates of Cats

While the proportion of owned cats that are micro-chipped (64%) is lower than dogs (76%) in Australia [44], the large difference in reclaim of cats in our study likely reflects the large number of stray cats which are unowned, which has been estimated to equal 1/3rd to 2/3rds of the owned cat population [45,46]. Semi-ownership of cats has been a long-standing issue and continues to play a role in the low reclaim rates for cats. An Australian internet survey found that 9% of respondents fed (daily) a cat that they did not perceive they owned [45], and another Australian study with a higher participation rate of males found 3% of respondents provided food daily to an unowned cat [47]. A feature distinguishing semi-owners from owners was that only owners actively acquired their cat, and passive acquisition was characteristic of semi-owners, who usually did not have their cat micro-chipped or sterilized [48].

In addition to the impact of semi-owned cats on the low micro-chipping rates of unowned cats, many owned cats are passively acquired [49]. A 2016 survey of cat owners found that 15% of owners sourced their cat from the street (i.e., cat had been a stray) [23]. In addition, 52% of cat owners reported that they acquired their cat for free. These cat owners may be less likely to pay a fee for registration and micro-chipping for cats acquired passively or for free, or may not be even aware of their pet’s microchip status.

The low reclaim rate of cats needs to be addressed in two ways. Firstly, there is obviously scope for councils to increase the number of micro-chipping events, and even to consider micro-chipping animals in the field as done by some counties in the USA (personal communication K. Peterson, Salt Lake County Animal Services, USA). However, to increase the proportion of cat owners attending these, secure areas need to be provided to prevent cats escaping, as well as transportation to the event for those without access to transport for their pet. An accompanying emphasis upon compliance monitoring for cats may motivate participation in such events. Secondly, initiatives are required to increase the proportion of semi-owned cats that are sterilized and micro-chipped by the carers who regularly feed a cat but do not perceive themselves as owners, and these are likely to center around free sterilization and micro-chipping targeted to areas of high cat intake.

### 4.3. Strategies Used by Councils to Reduce Euthanasia: Increasing Animal Rehoming Rates

The mean proportion of unclaimed dogs rehomed (71%) was higher than for cats (45%) and was higher than reported in a recent study (60% for dogs) using 2012–2013 DAM Plan data for Victoria [50]. Councils with low euthanasia for both dogs and cats, had significantly higher adoption rates for unclaimed animals—again indicating that council based factors have significant effects upon live release rate.

Strategies to maximize adoption also need to include maintaining the health of animals in the shelter, and vaccination is a fundamental part of any shelter preventative health program [51]. However, only one full-service council routinely vaccinated animals and this was within 48 h of entry when the veterinarian was available, and one hold operation vaccinated dogs on entry for parvovirus. The high density of animals in shelters and pounds, combined with admission of animals of unknown health status, increases the risk of exposure to infectious disease, and the increased stress experienced by the animals decreases their immune response to it [51]. This includes viral diseases with high morbidity and mortality, such as parvovirus in dogs, and highly infectious diseases such as cat flu or panleukopenia, which often result in euthanasia [51]. Importantly, a US study found that only 36% of dogs entering an animal shelter had protective antibody titers against canine distemper and parvovirus—both vaccine preventable diseases [52]. Of those pounds vaccinating animals, half only did so if the animal was expected to stay at the pound longer, or after the mandated holding period. To prevent an outbreak of infectious disease, vaccination prior to admission, or immediately on admission [51,53], is advised as immunity develops in unvaccinated dogs within hours after administration [54].

Foster programs have the potential to increase the adoptability of fearful, timid or boisterous animals through socialization and retraining, and staff interviewed commented that in their experience, foster programs were very effective. They can also provide treatment for animals with short-term health problems which would normally reduce adoptability, such as skin conditions including ringworm in cats, and provide post-operative care. A well-managed foster program can also help reduce the pressure on pound resources at busy times including Christmas and kitten season [55]. The Seattle Humane Society demonstrated the value of foster volunteers, not only in saving lives, but also saving their facility $US1.6 million in staff time to care for the 3001 animals that were fostered in the previous year [56]. Utilization of temporary foster care markedly improved odds of live release for dogs, and resulted in a 70% reduction in the prevalence of major or minor health or behavior concerns compared to before the dogs went out to foster [57].

Other activities that characterized pounds with high adoption rates were regular advertising in newspapers and on TV, and the provision of ‘home-like’ interaction areas for adopters where they could they observe the animal’s behavior in a non-shelter situation, and introduce their current pets to the animal under consideration. Many (70%) councils encouraged the finders of stray animals to adopt them if unclaimed. Finders of lost animals often form a bond or attachment to the animal, and are prepared to keep or formally adopt them in the event that the owner cannot be found [58].

### 4.4. Beliefs about Achieving Zero Euthanasia of Treatable and Adoptable Animals

Respondents were asked how long they thought it would take their council to get to zero euthanasia of adoptable and treatable animals, if they were provided with adequate resources. More respondents felt this was more achievable in the next 10 years for dogs (66%) than cats (40%), but 17% respondents felt it was never achievable for dogs, and 29% for cats. Even in the best performing councils, other strategies are needed to reduce euthanasia of healthy and treatable cats to zero.

### 4.5. Reducing the Euthanasia of Urban Stray Cats

#### 4.5.1. Behavioral Assessment of Unowned Cats

Many councils separately listed cats deemed as “feral” and nearly all were euthanized. When questioned about why and where the cats were caught, councils uniformly indicated they were trapped in response to complaints or calls from members of the public, and were trapped in inhabited areas. Usually they were assessed as feral based on un-socialized behaviour on the day they were trapped, often while still in the trap cage, and euthanized. However, research shows that suitability of cats for adoption cannot be determined for at least three days; and when stressed, pet cats can respond with more “feral” behaviours than stray cats [59].

Feral cats are best defined for purposes of management as cats which receive no food or shelter from humans, and are remote from human habitation. Based on this definition, none of the cats described by council staff as feral and euthanized fitted this definition. RSPCA Queensland decreased the proportion of cat admissions deemed as feral based on un-socialized behaviour and lack of identification from 8% to less than 1% by assessing cats’ sociability over three days instead of one, and utilizing foster carers to increase sociability of shy cats [30]. Appropriate time for assessment of suitability for adoption is recommended to decrease the unacceptably high euthanasia rates of these cats, and it is recommended that this be mandated. In most of Australia, cats deemed “feral” can be euthanized immediately without undertaking a minimal holding period. While it is recognised it is extremely stressful for a truly feral cat to be held, as a result of this legislation, thousands of potentially adoptable cats are euthanized without proper assessment.

#### 4.5.2. Attitudes towards Trap–Neuter–Return Programs for Cats

Urban stray cats make up approximately 80–90% of council pound admissions [60], and 50–70% of cat admissions to the RSPCA [30,35]; and they have significantly poorer outcomes in our study, and other studies, compared to dogs. Disturbingly, the worst quartile of councils was killing up to 98% of all cat admissions.

Trap–neuter–return programs are currently illegal in Australia and controversial in many countries [61,62,63]. These programs trap stray cats and kittens from urban locations that are overrepresented in shelter admissions, then neuter and return them to their site of origin. These programs have been shown to be very effective in reducing cat admissions and euthanasia in the USA, and cat-related complaints, provided high sterilization rates are achieved [14,15,16,64,65]. Faster declines in colony size occur when high adoption rates (30%) are achieved over the first two years [60]. In Montana, TNR resulted in a 36% decline in cat intake, an 87% decline in euthanasia, and an 84% decline in cat-related complaint calls [66]. In Texas, there was a 90% decline in cat-related complaint calls; and in Kentucky, a 51% decline in cat intake in targeted areas, compared with only a 20% decline in the entire service area [66]. In Florida, 60 stray cats/1000 residents, which was estimated to comprise half the stray cat population in that zip code, were sterilized over a two-year period, and adoptable cats were rehomed. Intake into the shelter from the targeted zip code subsequently decreased from 13 cats per 1000 residents to four per 1000 residents in two years [14]. However, in some reports of TNR programs, cat numbers did not decrease, and even increased, and the reasons for failure include not sterilizing most female cats, and failure to promptly manage immigrated or dumped cats by sterilization or adoption [67,68,69].

Despite respondents being informed that overseas, TNR programs had some success in reducing shelter intake of cats and euthanasia, and were provided with the URL describing a program in USA, more than half (54%) of pound managers in our cohort did not believe TNR would be effective in urban areas of Australia, and only 40% believed it would be effective. Concerns expressed related to the cats’ welfare, the magnitude of the cat population making it impossible to successfully reduce cat numbers, and negative effects on wildlife. The Australian Veterinary Association also does not support TNR programs because they state it is usually not possible to control the entry of new cats into the colony, and the long term welfare of the cats is generally very poor [70]. Additionally, they state that unowned cats have a serious impact on Australian wildlife, and can be the cause of considerable community nuisance, whether or not they have been sterilized. These reasons are generally not well supported by recent literature. For example, if immigrant and dumped cats are appropriately managed by sterilization or rehoming, colony size decreases over time [71]. Opposition based on concern for welfare of cats in TNR programs is not supported by research, because the welfare of these cats is not different from pet cats [49]. Euthanasia for health reasons is typically less than 1% of trapped cats, and the annual death rate in a managed colony is similar to that reported for pet cats [71,72].

Concerns regarding TNR in Australia frequently relate to the effect of cats on native wildlife, and 77% of our survey cohort voiced this concern. The effects of domestic cats on native wildlife are emotive and controversial, and there is evidence that depending on the location and native species involved, cats can have a negative, neutral or positive effect on native wildlife populations. It is widely accepted and supported by scientific evidence that feral cats (defined as cats located remote from human habitation, and with no dependence on humans for food or shelter) have a negative effect on some native wildlife species in Australia [73]. However, there is a dearth of well-designed studies in urban areas, including from Australia, that have investigated impact of urban cats on native wildlife.

Evidence supporting or contradicting claims that domestic cats are negatively affecting urban native animal species depends on the study area, native species involved, and the study methods used. Two studies which suggest cats have no impact on native populations of mammals or birds in urban areas of Australia come from Perth (Western Australia). One study examined three bushland sites; two that had either banned cats or required them to be confined at night and wear a bell during the day, and one adjacent to an unregulated cat area [74]. The study found the unregulated cat area to contain similar populations of medium-sized mammals to the other two sites, and the highest population of smaller mardo *Antechinus flavipes*, a species particularly susceptible to cat predation. A 2018 study from Melbourne, Australia found the abundance of the southern brown bandicoot to be higher in peri-urban areas compared with nature reserves, and was highest at sites with the most urbanized surroundings where cats were also prevalent, compared to the lower abundance of bandicoots in nature reserves where cats were largely absent [75]. These findings are consistent with those from Albany, New York, where no link was found between cat density and local small animal populations in a suburban nature reserve [76]. Another Perth study investigating 57 locations failed to find a link between cat density and passerine bird abundance and diversity, instead finding that bird populations were negatively affected by increasing density of housing, distance from bushland, and reduced size of bushland [77]. Both Perth studies concluded that habitat degradation and destruction, and not cats, negatively affected urban native wildlife populations. A recent report of prey observed to be captured by owned cats in Australia found that cats caught more mammals than other types of prey, followed by reptiles (small lizards) and then common birds. The mammals caught consisted almost exclusively of mice, rats, and rabbits [78].

In contrast, other studies suggest cats do have a negative effect on some mammal, reptile and bird species in urban and peri-urban areas. For example, the decline in the Australian mammal, the eastern barred bandicoot, at a municipal tip site was contributed to by cat predation of juveniles, along with road death of adults [79]. In one urban property in Perth, a single pet cat was suspected of exterminating a population of lizard (*C. fallens)* over two years, with a rapid decline observed in the first few months after the cat was introduced, and repopulation occurring after it was removed [80]. Other studies using modelling and based on observed predation by a limited number of cats, postulate that cat predation and other cat-related effects on reproduction exacerbate human-related effects [80]. A modelling study from the United Kingdom, based on current average urban cat densities, calculated that low level predation by domestic cats of urban songbirds, if compounded by non-lethal effects of cats on song-bird fecundity, would contribute to a decrease in species abundance, even to the point of extinction [81]. These non-lethal effects are the result of a ‘fear’ effect in suppressing reproduction. Another modelling study from New Zealand utilizing data on predation by 144 domestic cats in a 12-month period, calculated that cats have significant impacts on prey populations, particularly birds [82]. However, the estimates of bird predation by cats were more than the total urban population size of the bird species of interest, demonstrating the complexities of using modelling to accurately estimate population effects. This is particularly true when other effects are not included in the model, such as vegetation type and density, and the effect of cat predation on other bird predators, such as rats. For example, bird predation by rodents was not considered in the model, although rodents are a significant cause of bird predation in New Zealand, and rodents form a major part of cat prey [82]. In an Australia study of urban bushland sites, nest predation decreased as cat density increased, and this was hypothesized to reflect the positive effect of cat predation on other nest predators, such as rats [83].

Clearly well designed studies investigating the effects of differing cat densities on native species of interest are required in urban areas, and the results are likely location and species dependent. Nevertheless, where there is a negative effect of cat predation on native wildlife populations in urban areas, reducing the density of free-living urban cats using targeted community-based cat sterilization programs, such as TNR, that achieve high sterilization rates, would be expected to be beneficial for native wildlife.

There is debate whether cat predation of birds represents principally a compensatory or additive effect on bird populations [81]. Two studies from Europe (UK, France) found birds killed by cats in urban areas were generally less healthy than birds killed by cars or by flying into windows. Both authors also concluded that cats are opportunist hunters and tend to remove sick, old birds, and those which have fallen out of the nest (compensatory effect), rather than healthy birds (additive effect) [84,85]. In general, most of the species of birds killed in Australia by cats have an average life span of less than five years, meaning that at least 20% of the population are dying each year from a variety of causes including infectious, neoplastic and traumatic causes [78]. Collectively, these results suggest that the perceived negative effects of cats on native wildlife populations in suburban areas is overstated [86]. However, further well designed studies are required because the current research does not exclude the possibility that cat predation of native wildlife in urban areas may have significant effects on individual species in certain locations. That said, compared to results achieved by low level killing of cats, an effective TNR program would be expected to reduce the effect of cats on wildlife due to a greater overall reduction in cat numbers [15,16,60,65,71,87]. For example, in two Australian studies involving several cat colonies managed by TNR, cat numbers decreased by 31% in two years [60], 25% in 18 months and 57% in five years [71]. Of note, despite the number of reports in the literature of effective TNR programs, the authors found no reports in the literature of effective trap and kill programs in urban areas of Western countries. Given the number of urban stray cats euthanized, and the negative impact on mental health of workers involved, a trial of TNR is warranted to evaluate the effect on shelter intake and euthanasia of cats, and on native wildlife.

The risk of spread of disease to humans, wildlife and pet cats has also been voiced by opponents of TNR, and by our respondents. A number of diseases are of concern, including toxoplasmosis, ringworm, bartonella, tularaemia, plague, murine typhus and rabies [88,89]. Most are spread by direct contact and fleas, except toxoplasmosis, and rabies does not occur in Australia. Contrary to concern expressed by respondents in our and other studies, there is a low risk of disease transmission from cats to humans [90], and for most diseases, the risk of transmission is even lower from stray cats due to the lack of direct contact with the general public. Diseases transmitted from cats are much more likely to come from pet cats who are more frequently in direct contact with humans.

Toxoplasmosis is a disease of concern to humans and wildlife [91,92,93]. However, environmental contamination with infectious toxoplasmosis oocysts would be expected to be lower in sterilization programs compared to culling programs. In sterilization programs, mature cats are returned to their home location, creating colonies of older cats, many of which are immune to further toxoplasmosis infection. Typically, cats are only infected once in their life, usually when young, and after infection, shed oocysts for two to three weeks [91,93]. After infection they become immune, and rarely shed oocysts again. In contrast, in culling programs, new immunologically susceptible kittens are continuingly being born, become infected and shed toxoplasmosis oocysts, which are infectious within two to three days in the environment. In addition, naïve cats under a year of age shed more oocysts than older naïve cats when infected [92]. Sterilization programs also decrease the risk of spread of disease to pet cats, such as feline immunodeficiency virus (FIV), and reduce fight wounds because fighting and roaming in sterilized cats is reduced [69,94].

Although current Australian law prevents TNR except with a permit, if permission was gained to conduct these programs in areas contributing to high intake of stray cats into pounds, it could be a major advance in reducing euthanasia of healthy cats in pounds and shelters in Australia, and potentially also benefit native wildlife by decreasing numbers of stray cats. Ideally, this hypothesis should be tested by piloting such programs in controlled areas.

Importantly, and perhaps counterintuitively, trapping and killing unowned cats may lead to an increased cat population, rather than a decreased one (i.e., the opposite effect from that expected). This paradoxical effect on the target population has been observed in other species [87]. In a recent Tasmanian study, cat numbers tripled (increased between 75% and 210%) during the period 30% of cats were trapped and killed, but stabilized to previous numbers once the program stopped [87]. Although most of the trap sites were forested areas, one was a peri-urban area close to two waste management sites. Therefore, the low-level trapping and killing of urban stray cats as performed by most councils in Australia, may be counter-productive. Not only is it costly and has no medium or long-term effect in reducing cat numbers [95], it may increase cat numbers in problem areas, and therefore, represents poor use of council funds. It is strongly recommended that research trials are conducted in Australia to determine the efficacy of sterilization programs in reducing cat intake, euthanasia, and stray cat numbers in urban areas. This is most likely to be evident in high intake locations where there is sufficient budget to provide free sterilization for approximately 60 cats/1000 residents [14].

#### 4.5.3. Shelter–Neuter–Return Programs for Cats

Recently, shelter–neuter–return (SNR) programs where unadoptable, unidentified or unclaimed stray cats are neutered, ear-tipped and returned to within 300 m of their place of origin, were demonstrated to have markedly reduced euthanasia for stray and feral cats. In San Jose, USA, over four years, an SNR program in the council pound decreased euthanasia from over 70% to 23%, and 97% of healthy stray and feral cats were saved (10,080 cats over four years), when coupled with an existing TNR program. Cat and kitten admissions also decreased by 29%, euthanasia in the shelter from upper respiratory disease declined 99%, and dead cat pick up off streets declined by 20% [12]. In Australia, further research is needed in this area to determine its feasibility for reducing cat euthanasia. The number returned is small per 1000 residents, so would not be expected to result in a detectable increase in the number of free-roaming cats, but has a major impact in decreasing euthanasia.

### 4.6. Transparency of Data by Councils

This study was facilitated by the Victorian legislative requirement that councils develop a Domestic Animal Management plan every four years which “must outline programs, services and strategies the council intends to pursue in its council district to address overpopulation and high euthanasia rates for dogs and cats” [18]. Although not mandated, 70 of the 79 (89%) of Victorian councils reported (or provided their statistics via personal communication) for intake and outcomes for dog and cat management. Such transparency is unique in Australia, and it is strongly recommended that other states follow this example, and that annual reporting of data is mandated.

Transparency is part of the community engagement process, and facilitates resources being targeted to areas of need to improve outcomes for dogs and cats. In the year this study was undertaken, two councils euthanized ≥70% of unclaimed dogs and at least 26 councils euthanized ≥70% or more of unclaimed cats, seven of which were euthanizing ≥95%. For some councils, the high euthanasia rates resulted in over 1000 cats being euthanized, which can place a huge emotional strain on those involved. Transparency of these figures is needed to ensure councils provide explanations for why this is the case. Follow-up phone calls confirmed that these were largely urban strays, rather than truly feral cats remote from human habitation, and typically cats were not provided adequate time for assessment of suitability for rehoming [59,96]. In the DAM Plans of some councils, it was noted 100% of surrendered animals were euthanized, without providing an explanation for why this was the case.

A lack of consistency with the data in the council DAM Plans also prevents its use in broader animal management related research. For example, some councils did not provide rehoming and euthanasia statistics, and some do not include feral or owner-surrendered animals in their published data. Furthermore, for the DAM Plan data to be useful for further research, a more structured approach is needed to ensure the data is accurate and comparable across councils. In some cases, DAM Plans were updated with yearly data with all intake and outcome statistics, and for others, these only included one year of data or an average of the previous four years. In addition, nine councils did not make complete intake and outcome data available on their website or in their DAM Plan, and they not provide the data for this study after numerous requests for information. The Victorian government ‘Know Your Council’ website is a good platform for transparency of data, but was very limited in what was provided, with only numbers of animals registered and proportion reclaimed reported, but no intake or other outcome data.

The lack of effective data reporting in animal shelters is a well-documented phenomenon that has existed for decades [13,97]. At the time of writing, the only major Australian welfare organizations which report statistics relating to animal intake, reclaim, rehoming and euthanasia were the RSPCA, Lost Dogs Home, Cat Protection Society Victoria, Cat Haven and Animal Welfare League, Queensland.

Inadequate data hampers efforts to identify the dynamics, and associations and causes of euthanasia in animal shelters [98]. In the USA, attempts were made to collect extensive national data to quantify pet overpopulation and euthanasia, a costly and arduous task which did not achieve its aim of collecting all national data [13]. More recently, the Shelter Animals Count was formed in the USA to achieve this. This is a collaborative effort aimed at all sectors of the animal welfare community to create standardized reporting and definitions. Its aim is to promote transparency through disclosure of data to increase lifesaving opportunities [25].

In 2004, 18 local, regional and national animal welfare leaders in the USA gathered to approach the pet overpopulation issue. The Asilomar Accords were endorsed, and are a set of guidelines, definitions and outlines for a standardized data collection system. This framework has provided uniformity and the necessary standardization to track shelter populations. Since its development, over 76 counties and their individual welfare and animal control organizations have implemented this recording system [25]. Using this as a model, Australia could implement its own standardized reporting system to pave the way for transparency of all Australian animal pound and shelter data.

### 4.7. Limitations of the Study

The limitations of this project include that not all councils had data available, and the moderate participation response of 44% to our survey, which could lead to bias. Although it might be expected that better performing councils may have been more willing to share information, there were minimal differences in performance metrics between the survey and state cohorts. Councils from both metropolitan and regional areas were targeted to ensure that concerns and difficulties that are particular to their council areas were included. Reasons given for non-participation were time constraints, lack of knowledge as animals were immediately transferred or an unwillingness to participate.

As the survey cohort was only 35 councils, the sample sizes based on operation type were not ideal, and ranged from only 4–25, limiting comparisons between operation types. In addition, although we intended to statistically compare results of intake and outcome data of the survey cohort with policies and procedures, only six of the 35 councils in the survey cohort were full-service, and the others transferred animals for rehoming either immediately or after the mandated holding period. Because the data set was so small for full-service operations, we chose to describe their practices and where relevant, mention their outcome data.

## 5. Conclusions

Victorian councils have achieved substantial progress towards zero euthanasia of dogs, with 74% having achieved a euthanasia rate of ≤10% of intake, based on self-reported data for each council provided in their Domestic Animal Management plan or via personal communication. Notably, the best performing quartile of councils have achieved euthanasia percentages of 0–3% of dogs, while the poorest performing quartile euthanized 11–40%. By contrast, only one council achieved a euthanasia rate of ≤10% of admissions for cats. The quartile with the lowest euthanasia rates for cats achieved euthanasia rates of 7–28%, while the upper quartile for euthanasia were euthanizing 67–98% of cats. For both dogs and cats, there were no differences in outcomes between councils that ran their entire pound operation or those transferring to welfare agencies, either immediately, or after the minimal holding period of eight days in Victoria, although there were differences between demographic areas. Reclaim rates were high, with a median of 77% of dogs and 10% of cats reclaimed, and the upper quartile achieved 87–100% for dogs and 17–59% for cats. The median percentage of unclaimed animals rehomed was 72% for dogs and 49% for cats.

The ability to evaluate strategies for their effectiveness, leading to significant reductions in euthanasia of treatable and adoptable animals, are unlikely to occur without transparency of information, therefore this must be a priority for reducing euthanasia in Australia. In Australia and the USA, it has proven difficult to obtain consensus and participation in such data collection without strong motivation. In the USA, often this motivation has come from funding agencies, such as Maddie’s Fund, who require grant recipients to produce such information. Australia does not have a large philanthropic funding base available, so it is likely that to effect change, legislation will be required, as has occurred in Victoria. Using the Victorian model throughout Australia, including use of standardized definitions, would facilitate identification of proven and effective strategies to decrease euthanasia and guide deployment of financial (and other) resources in councils, whilst enabling evaluation of their efficacy. The public would also gain a better understanding of the issues, if this information was made available on a centralized website.

Euthanasia rates for cats are still unacceptably high, and the ramifications for damage to human mental health need to be considered. Existing literature strongly supports the importance of targeted sterilization programs for unowned cats in reducing euthanasia when high sterilization rates are achieved. As most of the studies come from overseas, it is strongly recommended that pilot programs in multiple locations in Australia are conducted as a matter of urgency to evaluate their efficacy.

Councils that transfer animals to other facilities can reduce euthanasia by including salient KPIs in their contracts. This would apply pressure on the welfare agencies to improve their outcomes, and make them more accountable for the proportion euthanized. However, welfare agencies that do not charge councils the true costs for rehoming animals they manage for them, do not provide incentives to councils to invest in strategies to reduce intake.

This study identified that some councils perform better than others in terms of outcomes for their animals, and that they use a variety of strategies with varied effectiveness. For example, councils with low euthanasia rates also have high reclaim and adoption rates, and typically engage in more strategies to increase registration and micro-chipping rates in their community, and involve community groups in their rehoming efforts.

## Figures and Tables

**Table 1:** Human and animal population and registration information for councils in the state (*n* = 79) and in the survey cohort (*n* = 35), obtained from Domestic Animal Management (DAM) Plans, council websites or via personal communication using data for the year 2016–2017 where available (or 2015–2016).

	All Councils in State (*n* = 79)					Survey Cohort of Councils (*n* = 35)				
Statistic	Mean	SD	Median	Lower Quartile	Upper Quartile	Mean	SD	Median	Lower Quartile	Upper Quartile
Residents ^1^	78,207	72,449	45,426	2904–16,066	139,511–313,521	90,439	72,794	74,329	2904–33,317	139,511–313,521
Households ^2^	29,956	26,193	17,737	1407–6699	54,556–102,334	35,259	26,772	31,523	1407–15,089	55,135–102,334
Socioeconomic status ^3^	5.6	2.8	6.0	1.0–3.0	8.0–10.0	5.9	2.9	6.0	1.0–3.0	8.0–10.0
**Dogs**										
Population ^4^	15,641	14,490	9085	581–3213	27,902–62,704	18,088	14,559	14,866	581–6663	27,902–62,704
Registered	8723	7138	7499	613–2794	11,777–31,246	11,170	8306	10,617	613–4901	13,193–31,246
Registered/1000 humans	149.8	64.3	162.5	17.0–91.0	192.0–336.0	150.0	61.8	157.0	21.0–102.0	192.0–336.0
Registration ($) entire	133.20	37.20	127.50	58.50–111.00	153.00–285.00	142.10	43.10	130.00	58.50–114.50	170.00–285.00
Registration ($) sterile	41.90	9.90	41.50	15.00–36.50	50.00–74.50	43.70	9.70	41.00	19.50–38.00	50.00–74.50
**Cats**										
Population ^4^	12,513	11,592	7268	465–2571	22,322–50,163	14,470	11,647	11,893	465–5331	22,322–50,163
Registered	2797	2325	2084	111–712	4652–9627	3465	2523	3210	111–1603	5198–9627
Registered/1000 humans	42.6	15.6	42.0	4.0–33.5	50.5–81.0	41.5	14.0	41.0	11.0–34.0	50.0–81.0
Registration ($) entire	111.70	32.20	110.50	47.00–93.00	132.00–190.00	120.00	30.00	120.00	47.00–102.00	139.00–190.00
Registration ($) sterile	34.90	9.50	36.00	10.00–30.00	40.00–57.00	37.60	8.50	37.00	12.00–33.00	42.00–57.00

Notes: ^1^ Human population data from the Australian Bureau of Statistics 2016 Census.^2^ Household data from the Victoria State Government population and household projections [22]. ^3^ Socio-economic status based on 1–10 scale (with 1 being the highest level of socio-economic disadvantage and 10 being the lowest level of socio-economic disadvantage) [19]. ^4^ Estimated dog and cat population in 2016 calculated using 20 dogs and 16 cats per 100 residents [23].

**Table 2:** Type of animal management operation for each council demographic development type.

Service Type	Total	Urban Metropolitan Developed	Urban Fringe	Urban Regional Town/City	Rural Agricultural
**Full-service** (8 day hold period and rehoming)	17%, 6/35	9%, 1/11	20%, 1/5	15%, 2/13	33%, 2/6
**Hold** (transfer to welfare agency after 8 day hold period)	11%, 4/35	0%	0%	31%, 4/13	0%
**Immediate transfer** (to welfare agency immediately/within 48 h)	66%, 23/35	91%, 10/11	60%, 3/5	54%, 7/13	50%, 3/6
**Hold dogs & immediate transfer cats**	6%, 2/35	0%	20%, 1/5	0%	17%, 1/6
**Total**	**100%**	**31%**, **11/35**	**14%**, **5/35**	**37%**, **13/35**	**17%**, **6/35**

**Table 3:** Dog and cat admissions for councils in state (*n* = 70) and in survey cohort (*n* = 35/79) and percentages of animals reclaimed, rehomed and euthanized, using the latest data available for the year 2016–2017 (or if not available, 2015–2016) obtained from the Domestic Animal Management (DAM) Plan or website of each council, or via personal communication.

	State (*n* = 70/79) #					Survey Cohort (*n* = 35/79)				
Statistic	Mean	SD	Median	Lower Quartile	Upper Quartile	Mean	SD	Median	Lower Quartile	Upper Quartile
**Dogs**										
Total intake	427	423	277	6–129	554–2196	551	488	399	6–192	837–2196
Intake/1000 humans	7.5	5.5	6.5	0.7–3.2	10.4–24.6	7.2	4.7	6.6	0.7–3.6	10.6–20.5
Proportion reclaimed	73.1	17.2	77.4	32.6–61.0	86.8–100.0	73.6	18.0	80.8	32.6–59.8	86.8–100
Proportion rehomed	19.0	13.0	15.8	0.0–9.1	26.9–52.1	19.7	14.0	16.4	0.0–8.8	28.5–52.1
Proportion euthanized	8.0	7.7	6.2	0.0–2.6	10.6–40.2	6.6	5.5	4.8	0.0–2.1	10.6–22.1
Proportion unclaimed rehomed *	71.3	16.3	71.7	21.1–62.5	83.6–100.0	73.9	11.6	74.8	50.0–66.7	83.6–91.5
Proportion unclaimed euthanized *	28.7	16.3	28.3	0.0–16.4	37.5–78.9	26.1	11.6	25.2	8.5–16.4	33.3–50.0
**Cats**										
Total intake	429	502	203	0–127	567–2182	490	496	245	3–132	777–2182
Intake/1000 humans	7.6	6.0	6.5	0.0–2.2	10.5–27.6	6.5	5.2	5.7	0.5–1.7	9.2–20.6
Proportion reclaimed	12.6	10.2	9.8	0.0–5.4	17.1–59.4	14.3	8.9	12.6	0.0–7.8	19.5–33.3
Proportion rehomed	39.2	22.6	38.8	0.0–18.8	59.1–81.3	44.1	19.0	51.7	7.7–24.8	59.6–69.8
Proportion euthanized	48.3	24.7	42.6	6.5–28.4	66.5–97.6	41.5	18.4	33.6	11.3–28.4	52.4–83.9
Proportion unclaimed rehomed *	45.2	25.7	48.8	0.0–24.0	67.4–91.3	51.3	21.1	57.6	8.4–29.0	67.6–85.5
Proportion unclaimed euthanized *	54.8	25.7	51.2	8.7–32.6	76.0–100.0	48.7	21.1	42.4	14.5–32.4	71.0–91.6

Notes: Statistics for total intake represent all dogs and cats admitted to councils, regardless of outcome. The proportion of each outcome (i.e., reclaimed, rehomed and euthanized) were calculated using an adjusted intake that excluded the following animals: Stolen/escaped; unassisted deaths/deceased on entry; and animals being processed/held or fostered at time of statistics publication and therefore would be accounted for in the following year of data). * Percentage of unclaimed animals that were subsequently rehomed or euthanized. # Complete intake and outcome data could not be obtained in the DAM Plan of the following councils, and they were not provided when requested: Baw Baw; Golden Plains; Hindmarsh, Horsham; Indigo; Maribyrnong; Melton; Moorabool; and Wyndham.

**Table 4:** Dog and cat admissions for each operation type in the survey cohort (*n* = 35/79) and percentages of animals reclaimed, rehomed and euthanized, using the latest data for the year 2015–2016 or 2016–2017 obtained from the Domestic Animal Management (DAM) Plan or website of each council, or via personal communication.

	Full-Service (*n* = 6)						Hold (Dogs: *n* = 6; Cats: *n* = 4)						Immediate Transfer (Dogs: *n* = 23; Cats: *n* = 25)						
Statistic	Mean	SD	Median	Lower Quartile	Upper Quartile		Mean	SD	Median	Lower Quartile	Upper Quartile		Mean	SD	Median	Lower Quartile	Upper Quartile		*p-*Value
**Dogs**																			
Total intake	493	390	311	155–188	793–1019	ns	531	384	389	116–302	872–1116	ns	569	544	408	6–192	837–2196	ns	0.967
Intake/1000 humans	8.6	4.4	9.0	2.5–6.3	12.2–13.2	ns	11.0	5.3	10.4	4.7–8.3	11.9–20.5	ns	6.0	4.2	5.1	0.7–2.1	9.2–15.6	ns	0.079
Proportion reclaimed	68.9	20.1	68.9	41.0–58.5	86.3–89.6	ns	74.0	17.0	81.5	43.1–65.1	85.7–86.8	ns	74.6	18.5	80.6	32.6–59.8	88.5–100	ns	0.794
Proportion rehomed	26.2	17.3	28.5	8.8–12.0	29.7–52.1	ns	19.4	13.9	14.1	9.9–10.1	22.0–46.1	ns	18.4	13.5	15.6	0.0–6.9	26.9–45.3	ns	0.488
Proportion euthanized	4.9	4.4	2.6	1.6–1.7	6.9–11.8	ns	6.7	4.5	5.7	2.0–3.1	10.8–12.8	ns	7.0	6.0	4.9	0.0–2.1	10.6–22.1	ns	0.632
Proportion unclaimed rehomed *	84.7	7.7	87.7	71.7–84.4	88.3–91.5	ns	73.7	12.4	76.6	55.3–63.2	80.9–89.7	ns	71.4	11.1	71.9	50.0–66.7	79.5–90.5	ns	0.062
Proportion unclaimed euthanized *	15.3	7.7	12.3	8.5–11.7	15.6–28.3	ns	26.3	12.4	23.4	10.3–19.1	36.8–44.7	ns	28.6	11.1	28.2	9.5–20.5	33.3–50.0	ns	0.062
**Cats**																			
Total intake	485	525	176	123–145	638–1343	ns	598	422	567	226–236	959–1030	ns	474	518	243	3–126	738–2182	ns	0.530
Intake/1000 humans	8.9	7.5	8.4	1.2–4.0	10.5–20.6	ns	11.0	5.9	9.4	6.2–6.5	15.4–18.9	ns	5.3	4.2	4.5	0.5–1.6	8.2–17.4	ns	0.098
Proportion reclaimed	11.1	6.7	9.7	3.4–6.5	17.0–19.0	ns	10.1	5.3	10.7	3.5–6.0	14.3–15.5	ns	15.7	9.6	13.2	0.0–8.6	21.7–33.3	ns	0.360
Proportion rehomed	49.5	17.0	60.8	24.8–38.6	61.3–62.0	ns	30.0	19.1	29.7	7.7–15.5	44.5–52.7	ns	45.3	19.0	52.1	11.3–33.3	59.1–69.8	ns	0.222
Proportion euthanized	39.4	22.2	32.7	19.0–21.7	51.7–71.8	ns	60.0	21.4	62.1	31.9–46.1	73.9–83.9	ns	39.0	16.1	33.5	11.3–28.4	50.8–81.1	ns	0.186
Proportion unclaimed rehomed *	56.8	21.9	65.0	25.7–42.8	73.9–76.6	ns	33.7	22.5	32.1	8.4–17.5	49.9–62.3	ns	53.0	20.2	61.4	14.2–43.7	67.6–85.5	ns	0.188
Proportion unclaimed euthanized *	43.2	21.9	35.0	23.4–26.1	57.2–74.3	ns	66.3	22.5	68.0	37.7–50.1	82.5–91.6	ns	47.0	20.2	38.6	14.5–32.4	56.3–85.8	ns	0.188

Notes: Statistics for total intake represent all dogs and cats admitted to councils, regardless of outcome. The proportion of each outcome (i.e., reclaimed, rehomed and euthanized) were calculated using an adjusted intake that excluded the following animals: Stolen/escaped; unassisted deaths/deceased on entry; and animals being processed/held or fostered at time of statistics publication and therefore would be accounted for in the following year of data). For comparison of operation types, medians with a different letter are significantly different (*p* < 0.05) from other medians in the row and ‘ns’ indicates not significant. *p* values were adjusted using Bonferroni correction. *Proportion of dogs or cats that that were not reclaimed by an owner that were subsequently rehomed or euthanized.

**Table 5:** Proportion of councils where final decision for feral cat euthanasia was made by council employees, veterinarians or shelter staff.

Service Type	Council Ranger/Staff	Veterinarian	Shelter
Full-service (*n* = 6)	83%, 5/6	17%, 1/6	N/A
Hold (*n* = 4)	75%, 3/4	25%, 1/4	0%
Immediate transfer (*n* = 23)	13%, 3/23	13%, 3/23	74%, 17/23
Hold dogs, immediate transfer (*n* = 2)	100%, 2/2	0%	0%
**Total**	**37%**, **13/35**	**14%**, **5/35**	**49%**, **17/35**

**Table 6:** Frequency of euthanasia for dogs and cats in full-service operations (*n* = 6).

	Dogs				Cats			
Euthanasia Reason	Never	Sometimes	Frequently	Always	Never	Sometimes	Frequently	Always
Too young	100%, 6/6	0%	0%	0%	50%, 3/6	50%, 3/6	0%	0%
Too old	67%, 4/6	17%, 1/6	0%	17%, 1/6	67%, 4/6	33%, 2/6	0%	0%
Behavioral issues ^1^	17%, 1/6	50%, 3/6	0%	33%, 2/6	0%	33%, 2/6	33%, 2/6	33%, 2/6
Insufficient space in facility	100%, 6/6	0%	0%	0%	67%, 4/6	33%, 2/6	0%	0%
Too many days in shelter	100%, 6/6	0%	0%	0%	100%, 6/6	0%	0%	0%
Non-life-threatening disease	100%, 6/6	0%	0%	0%	33%, 2/6	17%, 1/6	33%, 2/6	17%, 1/6
Life-threatening infectious disease ^2^	0%	40%, 2/5	20%, 1/5	40%, 2/5	0%	20%, 1/5	20%, 1/5	60%, 3/5
Health issue untreatable within budget/time allowed	67%, 4/6	33%, 2/6	0%	0%	33%, 2/6	33%, 2/6	17%, 1/6	17%, 1/6
Health issue requiring costly/long-term management ^2^	33%, 2/6	50%, 3/6	0%	17%, 1/6	20%, 1/5	40%, 2/5	20%, 1/5	20%, 1/5

^1^ Question did not specify whether this was aggression or general behavioral issues. For dogs, both “always” responses and 2/3 of “sometimes” responses stated that this was for aggressive, fear-biting, people-biting ones. For cats, trapped cats were not included, and were typically regarded as feral and euthanized. ^2^ In cases where proportions were out of five, one council left this up to the veterinarian to decide.

**Table 7:** Strategies utilized by councils in survey cohort (35/79) to reduce dog and cat intake.

Strategies
**Promoting responsible pet ownership**
Required by all councils under Domestic Animals Act 1994 Utilized local newspaper, social media, pet expositions, and school visits Provided training programs and information seminars
**Returning roaming animals directly to owners**
94% (33/35) of councils enabled officers (with some discretion) to directly return roaming animals with current registration (license) to their owner, rather than be impounded Minority returned unregistered (unlicensed) animals and had owner complete registration at point of return, and followed up for payment Greater number of councils returned unregistered animals after storms or fireworks
**Escalating fines for owners of repeatedly wandering animals**
89% (31/35) of councils allowed officers to use their discretion to issue a warning or a fine for wandering animals 97% (34/35) encouraged responsible pet ownership behaviors by utilizing escalating penalties for wandering animals, example, for initial offence, owners were issued a warning without financial penalty. Second offence, owners issued an infringement notice, and third offence, wandering animal was impounded
**Subsidized sterilization programs**
40% (14/35) of councils provided subsidized sterilization programs 63% (19/30) of respondents were aware of AVA vouchers available for subsidized sterilization for people on limited income (owner paid 67% of cost), but only 53% (10/19) distributed them 2/14 restricted sterilization programs to pensioners and concession card holders 1/35 offered free sterilization clinics for cats twice a year, mainly targeted to disadvantaged areas with high cat intake 2/21 without sterilization programs had special impound rebates ○ 1/21 if animal was impounded and entire, owner could return within a month and council would reimburse them with a portion of the impound fee if the animal was subsequently sterilized ○ 1/21 had arrangement for impounded entire dogs where, for a set price which included the release/impound fee, the animal was sterilized, and registered
**Offering alternatives to surrender**
57% (17/30) of councils utilized strategies to encourage owners to keep their animal and offered solutions 10% (3/30) offered free pound housing of the animal while the owner mended fences, or organized alternative methods of confinement 43% (13/30) provided advice to owners verbally or via pamphlets regarding behavioral issues and containment options 1/30 asked owners to consider their decision for another seven days before proceeding with surrender 10% (3/30) offered to help owners with fees (i.e., impoundment or sterilization fees) if this was the reason for surrender 2/30 held surrendered animals for three or eight days (respectively), in case the owner changed their mind. Note: A cooling-off period for owner-surrendered animals is not a legislative requirement in the Domestic Animals Act 1994
**Slowing intake when at or near capacity**
If a micro-chipped or otherwise identifiable, unregistered animal was found, it was returned to the owner and its registration status either followed up (*n* = 4) or completed online at point of return (*n* = 1) Three councils contacted volunteers, rescue organizations, and surrounding pounds and shelters, and attempted to place animals One council returned roaming animals free of charge to the owner (depending on history) One reduced the number of cat traps available to reduce intake One attempted to slow intake by referring people who lived outside the council to surrender to their own pound facility One advised those surrendering animals of the current situation when nearing capacity

**Table 8:** Strategies utilized by councils to increase animal reclaim rates.

Strategies
**Increasing animal identification**
17% (6/35) ran micro-chipping events to increase the number of animals with microchips and the accuracy of the owner contact details. One council targeted these to young and older pets 97% (34/35) conducted compliance monitoring to increase current registrations using “door knocking” 94% (32/34) targeted compliance monitoring to people where registration for an animal had not been renewed 12% (4/34) utilized microchip registry data to extract the details of owners of animals living in postcodes within that council, and identify those without current registration 12% (4/34) first informed owners via telephone, email, or SMS of the overdue renewal 1/34 advertised an amnesty period during which owners could register their animals without penalty, prior to door knocking being undertaken 24% (8/34) offered to check microchip details when they were door-knocking for compliance checks
**Advertising stray animals**
81% (13/16) used the council website, 4 used Facebook, 2 advertised in the local newspaper, 2 used rescue group websites, and 1 posted brochures at veterinary clinics and on a community app
**Extended holding and reclaim rates**
1/35 owners could pick up wandering animals over weekends and until 8:30 pm on weekdays 1/35 identified stray animals held at council offices until the evening, before being transferred to welfare agency, to facilitate contacting owners, and reclaim of animal

**Table 9:** Strategies utilized by councils to increase rehoming rates.

Strategies
**Vaccination protocols**
1/6 full service operations routinely vaccinated dogs and cats within 48 h depending on the veterinarian’s ability to attend the shelter All full-service (*n* = 6) and hold (*n* = 6) operations reported undertaking some of the following routine treatments when required: parasites (fleas, worms, mites, ringworm/mange), kennel cough (dogs), cat flu (cats), trimming nails, clipping matted fur, treating cuts/abrasions, wounds, treating animals in pain, and euthanasia if necessary.
**Encourage finders of stray animals to adopt**
70% (21/30) across full-service, hold and immediate transfer operations encouraged those bringing in/reporting strays to adopt them if unclaimed, and either recorded their expressions of interest on the relevant paperwork, or directed them to the shelter they were transferred to
**Programs in full-service facilities (*n* = 6)**
Animals available for adoption advertised on social media, council websites, newspapers 1/6 reduced adoption fees for older animals 2/6 provided free registration for the remainder of the year or for a year for animals adopted from facility 83% (5/6) worked in collaboration with rescue organizations 2/6 had volunteer programs and 2/6 were developing them ○ Volunteers were tasked with walking, grooming and feeding animals, and assessing their suitability for adoption ○ Volunteer programs were conducted in collaboration with a rescue group (*n* = 1), and involved school and special needs children, or (*n* = 1) individuals in corrective service (as part of their community service requirements), or on government unemployment benefits 50% (3/6) utilized fostering programs through rescue groups or veterinary clinics ○ 2/3 provided support in the form of food, bedding, litter, and veterinary treatments/medication ○ 1/3 rescue group received no support from the council but owned and ran their own “opportunity” shop, with proceeds used to fund their foster program 67% (4/6) accepted public donations of food, bedding, blankets, and toys but none ran appeals for funds 1/6 special training program for adoption staff 1/6 formal rehabilitation program to improve the adoptability of animals 1/6 utilized adoptions through local pet shops 1/6 utilized adoptions through local veterinary clinics 1/6 special induction/education building with one room set up with couch in home-like setting for prospective adopters to bring children and other pets to meet animals

**Table 10:** Frequency of animal transfers from full-service operations to other animal welfare organizations and reasons for transfer.

Factors Influencing the Transfer of Animals to Other Organizations?	Never/Not Applicable	Sometimes	Frequently	Always
A request for animals from a rescue group or other welfare agency	50% (3/6)	17% (1/6)	0%	33% (2/6)
To increase an animal’s chance of adoption	17% (1/6)	0%	17% (1/6)	67% (4/6)
Shelter is at full capacity	33% (2/6)	50% (3/6)	17% (1/6)	0%
Shelter has an existing relationship with specific breed organization	67% (4/6)	17% (1/6)	0%	17% (1/6)

**Table 11:** Proportion of responses in survey cohort (35/79) regarding the possibility of rehoming all treatable and adoptable dogs and cats and the timeframe that this could be achieved (categorized demographically and by operation type).

		Demographic Type				Operation Type		
Response	Total	Urban Metropolitan Developed	Urban Fringe	Urban Regional Town/city	Rural Agricultural	Full-Service	Hold	Immediate Transfer
**Question 1**	**Given resources, could council rehome all treatable and adoptable dogs?**							
Yes	86%, 30/35	82%, 9/11	100%, 5/5	92%,12/13	67%, 4/6	100%, 6/6	83%, 5/6	83%, 19/23
No	11%, 4/35	18%, 2/11	0%	8%, 1/13	17%, 1/6	0%	0%	17%, 4/23
Unsure/NA	3%, 1/35	0%	0%	0%	17%, 1/6	0%	17%, 1/6	0%
**Question 2**	**Given resources, could council rehome all treatable and adoptable cats?**							
Yes	40%, 14/35	45%, 5/11	40%, 2/5	46%, 6/13	17%, 1/6	33%, 2/6	50%, 2/4	40%, 10/25
No	49%, 17/35	55%, 6/11	40%, 2/5	46%, 6/13	50%, 3/6	67%, 4/6	50%, 2/4	44%, 11/25
Unsure/NA	11%, 4/35	0%	20%, 1/5	8%, 1/13	33%, 2/6	0%	0%	16%, 4/25
**Question 3**	**Provided adequate resources, zero euthanasia of all treatable and adoptable dogs could be achieved in?**							
Already	9%, 3/35	9%, 1/11	20%, 1/5	0%	17%, 1/6	50%, 3/6	0%	0%
<10 years	57%, 20/35	55%, 6/11	60%, 3/5	62%, 8/13	50%, 3/6	50%, 3/6	33%, 2/6	65%, 15/23
10–20 years	3%, 1/35	0%	0%	8%, 1/13	0%	0%	17%, 1/6	0%
20–30 years	0%	0%	0%	0%	0%	0%	0%	0%
30–40 years	0%	0%	0%	0%	0%	0%	0%	0%
Never	17%, 6/35	18%, 2/11	0%	23%, 3/13	17%, 1/6	0%	17%, 1/6	22%, 5/23
Unsure/NA	14%, 5/35	18%, 2/11	20%, 1/5	8%, 1/13	17%, 1/6	0%	33%, 2/6	13%, 3/23
**Question 4**	**Provided adequate resources, zero euthanasia of all treatable and adoptable cats could be achieved in?**							
Already	6%, 2/35	9%, 1/11	20%, 1/5	0%	0%	33%, 2/6	0%	0%
<10 years	34%, 12/35	27%, 3/11	20%, 1/5	38%, 5/13	50%, 3/6	17%, 1/6	50%, 2/4	36%, 9/25
10–20 years	11%, 4/35	9%, 1/11	0%	23%, 3/13	0%	17%, 1/6	0%	12%, 3/25
20–30 years	3%, 1/35	0%	0%	8%, 1/13	0%	17%, 1/6	0%	0%
30–40 years	6%, 2/35	0%	40%, 2/5	0%	0%	0%	0%	8%, 2/25
Never	29%, 10/35	36%, 4/11	20%, 1/5	23%, 3/13	33%, 2/6	17%, 1/6	25%, 1/4	32%, 8/25
Unsure/NA	11%, 4/35	18%, 2/11	0%	8%, 1/13	17%, 1/6	0%	25%, 1/4	12%, 3/25

Note: statistics for the two councils that held dogs and immediately transferred cats were separated into their respective dog and cat categories.

## Appendix A

**Table A1:** Human and animal population and registration information (including the price to register entire and sterile animals) for each demographic area in the state (*n* = 79), using the latest data available for the year 2015–2016 or 2016–2017 obtained from the Domestic Animal Management (DAM) Plan or website of each council, or via personal communication.

	Urban Metropolitan Developed (*n* = 23)						Urban Fringe (*n* = 10)						Urban Regional Town/City (*n* = 22)						Rural Agricultural (*n* = 24)						
Statistic	Mean	SD	Median	Lower Quartile	Upper Quartile		Mean	SD	Median	Lower Quartile	Upper Quartile		Mean	SD	Median	Lower Quartile	Upper Quartile		Mean	SD	Median	Lower Quartile	Upper Quartile		*p*-Value
Humans ^1^	147,367	48,882	148,039	87,355–11,606	170,093–313,521	c	129,698	78,107	148,531	2904–64,280	207,830–228,088	c	54,695	47,615	40,738	20,904–29,306	54,564–238,603	b	12,027	4770	12,079	3912–7428	16,051–19,817	a	<0.001
Households ^2^	56,885	14,668	55,135	34,840–44,794	65,131–102,334	c	44,589	25,721	52,726	1407–22,071	66,517–72,595	c	22,229	19,292	15,859	7507–12,445	22,392–96,065	b	5137	2016	5224	1744–3260	6680–8557	a	<0.001
Socioeconomic status ^3^	7.6	2.8	9.0	1.0–6.0	10.0–10.0	b	7.5	2.3	8.0	2.0–7.0	9.0–10.0	b	4.4	2.1	4.0	1.0–3.0	6.0–9.0	a	4.0	2.0	3.5	1.0–2.5	6.0–7.0	a	<0.001
**Dogs**																									
Population ^4^	29,473	9776	29,608	17,471–22,321	34,019–62,704	c	25,940	15,621	29,706	581–12,856	41,566–45,618	c	10,939	9523	8148	4181–5861	10,913–47,721	b	2405	954	2416	782–1486	3210–3963	a	<0.001
Registered	11,922	5278	11,227	3128–8388	15,068–27,218	c	14,635	10,081	14,995	613–5710	19,366–29,805	bc	9076	6313	7655	2549–5350	10,807–31,246	b	2354	898	2344	922–1947	2852–4207	a	<0.001
Registered/1000 humans	81.9	27.3	85.0	21.0–59.0	97.0–141.0	a	135.0	64.0	155.0	17.0–85.0	188.0–211.0	b	174.0	33.1	176.0	122.0–148.0	196.0–229.0	bc	204.3	48.2	190.5	137.0–178.0	213.0–336.0	c	<0.001
Price ($) entire	154.90	35.20	153.00	58.50–141.00	180.00–227.50	c	145.50	56.60	131.00	88.00–111.00	154.00–285.00	bc	132.60	24.40	129.50	80.80–116.00	150.00–182.90	b	108.70	23.80	107.80	60.00–94.50	121.80–170.00	a	<0.001
Price ($) sterile	50.00	10.30	51.00	19.50–47.00	55.00–74.50	c	40.70	5.70	40.30	31.80–37.00	45.00–50.00	b	40.96	9.26	41.50	15.00–38.00	44.00–57.00	b	35.50	5.70	35.80	22.00–31.00	40.50–43.50	a	<0.001
**Cats**																									
Population ^4^	23,579	7821	23,686	13,977–17,857	27,215–50,163	c	20,752	12,497	23,765	465–10,285	33,253–36,494	c	8751	7618	6518	3345–4689	8730–38,176	b	1924	763	1933	626–1189	2569–3171	a	<0.001
Registered	4453	1597	4613	1603–3292	5601–7421	c	4116	2888	4337	111–922	6497–8301	bc	2616	2103	2018	428–1419	3118–9627	b	639	330	570	208–440	728–1486	a	<0.001
Registered/1000 humans	31.0	9.2	33.0	11.0–24.0	39.0–47.0	a	33.7	14.0	34.5	4.0–27.0	43.0–53.0	a	47.9	11.6	49.0	20.0–44.0	51.0–66.0	b	53.8	15.0	50.5	34.0–43.0	66.0–81.0	b	<0.001
Price ($) entire	116.40	39.60	112.50	47.00–92.00	145.50–190.00	ab	95.40	30.40	91.00	54.00–82.00	111.00–143.10	a	126.30	22.20	126.00	79.30–109.00	143.20–171.00	b	99.70	27.90	100.60	48.00–79.00	115.50–170.00	a	0.010
Price ($) sterile	35.37	9.78	35.00	12.00–31.00	40.00–55.00	ab	31.21	9.56	28.00	19.70–23.00	39.00–48.00	a	38.47	10.22	40.00	10.00–36.00	43.00–57.00	b	32.87	7.64	33.70	16.00–27.25	38.50–50.00	a	0.029

NOTE: For comparison of demographic types, medians in the rows followed by a different letter (a, b or c) are significantly different in pairwise comparisons (*p* ≤ 0.05). *p* values were adjusted using Bonferroni correction. ^1^ Human population data from the Australian Bureau of Statistics Census 2016 data. ^2^ Household data from the Victoria State Government population and household projections [22]. ^3^ Socio-economic status based on 1–10 scale (with 1 being the highest level of socio-economic disadvantage and 10 being the lowest level of socio-economic disadvantage) [19]. ^4^ Estimated dog and cat population in 2016 calculated using 20 dogs and 16 cats per 100 residents [23].

## Appendix B

**Table A2:** Dog and cat admissions for each demographic in the state (*n* = 79) and percentages of animals reclaimed, rehomed and euthanized, using the latest data for the year 2015–2016 or 2016–2017 obtained from the Domestic Animal Management (DAM) Plan or website of each council, or via personal communication.

	Urban Metropolitan Developed (*n* = 23)						Urban Fringe (*n* = 10)						Urban Regional Town/City (*n* = 22)						Rural Agricultural (*n* = 24)						
Statistic	Mean	SD	Median	Lower Quartile	Upper Quartile		Mean	SD	Median	Lower Quartile	Upper Quartile		Mean	SD	Median	Lower Quartile	Upper Quartile		Mean	SD	Median	Lower Quartile	Upper Quartile		*p*-Value
**Dogs**																									
Total intake	441	331	341	99–220	554–1196	b	633	460	669	11–221	997–1279	b	671	519	474	188–304	872–2196	b	114	88	88	6–58	137–342	a	0.000
Intake/1000 humans	2.8	1.6	2.5	0.7–1.8	3.6–7.8	a	5.1	0.8	4.9	3.8–4.7	5.8–6.3	b	11.8	3.8	10.9	6.9–9.1	12.5–20.9	d	9.4	6.4	7.2	1.0–6.2	12.5–24.6	c	0.000
Proportion reclaimed	85.2	8.9	86.8	67.3–80.6	91.4–98.5	b	86.8	10.1	86.0	72.5–79.5	95.5–100	b	66.3	14.3	65.2	41.0–57.6	80.5–88.5	a	61.1	16.9	63.8	32.6–49.1	69.6–88.5	a	0.000
Proportion rehomed	10.5	6.7	8.7	1.0–5.6	14.5–25.0	a	9.6	7.3	11.0	0.0–2.9	14.2–20.9	a	25.4	12.0	24.6	9.4–14.0	31.7–52.1	b	25.6	13.8	26.2	3.8–14.3	37.0–49.6	b	0.000
Proportion euthanized	4.2	3.0	4.0	0.0–1.8	6.8–10.6	a	3.6	3.2	3.4	0.0–0.9	5.6–9.4	a	8.3	4.6	8.0	1.3–4.9	11.5–17.7	b	13.3	10.9	11.8	2.2–4.4	16.7–40.2	b	0.000
Proportion unclaimed rehomed	71.0	14.1	67.5	43.8–61.4	81.3–100	ns	76.2	14.0	76.1	59.5–63.4	87.7–100	ns	75.1	10.8	74.9	55.3–67.5	83.6–90.5	ns	66.4	22.0	67.2	21.1–57.6	85.2–92.9	ns	0.581
Proportion unclaimed euthanized	29.0	14.1	32.6	0.0–18.8	38.6–56.3	ns	23.8	14.0	23.9	0.0–12.3	36.6–40.5	ns	24.9	10.8	25.1	9.5–16.4	32.5–44.7	ns	33.6	22.0	32.8	7.1–14.8	42.4–78.9	ns	0.581
**Cats**																									
Total intake	452	474	248	78–159	567–2126	b	664	773	382	13–69	1,18–1962	ab	632	536	420	143–243	1030–2182	b	132	101	123	0–90	156–496	a	0.000
Intake/1000 humans	2.8	2.3	1.9	0.7–1.3	4.0–10.3	a	4.4	3.1	4.3	1.1–1.5	6.5–9.4	a	10.8	4.5	10.1	5.3–6.7	12.9–20.6	b	11.1	6.8	9.5	0.0–7.2	15.1–27.6	b	0.000
Proportion reclaimed	17.3	8.2	17.4	3.0–8.9	21.1–31.4	c	19.8	19.2	19.4	0.0–3.7	26.6–59.4	bc	10.3	4.3	9.8	3.5–6.6	13.0–19.5	b	6.6	7.4	3.6	0.7–1.9	9.1–33.3	a	0.000
Proportion rehomed	41.0	21.5	40.6	11.3–20.6	62.0–73.0	ns	40.2	22.7	44.8	9.4–18.3	59.7–67.0	ns	46.3	15.6	47.2	7.7–36.2	58.5–71.3	ns	30.0	27.5	21.8	0.0–4.2	56.8–81.3	ns	0.210
Proportion euthanized	41.7	22.1	31.7	6.5–23.9	65.8–76.4	ns	40.0	21.7	37.0	11.3–26.5	49.3–82.9	ns	43.5	15.9	40.7	21.2–31.9	51.7–83.9	ns	63.5	29.7	72.7	16.3–32.1	93.1–97.6	ns	0.058
Proportion unclaimed rehomed	49.7	25.7	57.0	14.2–25.5	71.0–91.3	ns	48.9	24.9	51.3	15.4–26.1	67.7–85.5	ns	51.6	17.2	53.7	8.4–42.5	64.7–77.1	ns	32.7	29.8	23.0	0.0–4.4	60.9–83.3	ns	0.122
Proportion unclaimed euthanized	50.3	25.7	43.0	8.7–29.0	74.5–85.8	ns	51.1	24.9	48.7	14.5–32.3	74.0–84.6	ns	48.4	17.2	46.3	22.9–35.3	57.5–91.6	ns	67.3	29.8	77.0	16.7–39.1	95.6–100	ns	0.122

Notes: Statistics for total intake represent all dogs and cats admitted to councils, regardless of outcome. The proportion of each outcome (i.e., reclaimed, rehomed and euthanized) were calculated using an adjusted intake that excluded the following animals: stolen/escaped; unassisted deaths/deceased on entry; and animals being processed/held or fostered at time of statistics publication and therefore would be accounted for in the following year of data). For comparison of operation types, medians with a different letter (a, b or c) are significantly different (*p* ≤ 0.05) from other medians in pairwise comparison and ‘ns’ indicates not significant. *p* values were adjusted using Bonferroni correction.
